# Monoclonal antibodies targeting PCDH7 inhibit tumor growth and enhance immune responses in *KRAS*-mutant non–small cell lung cancer

**DOI:** 10.1126/sciadv.aeb0794

**Published:** 2026-06-03

**Authors:** Nicole Novaresi, Poorva Ghosh, Shayna Thomas-Jardin, Hui Deng, Xuejun Fan, Zhiqiang Ku, Wei Xiong, Xiaorong Zhou, Jingfei Zhu, Huiyu Li, Mahesh S. Padanad, Bethany Smith, Chul Ahn, John D. Minna, Zhiqiang An, Ningyan Zhang, Kathryn A. O’Donnell

**Affiliations:** ^1^ Department of Molecular Biology, UT Southwestern Medical Center, Dallas, TX 75390, USA.; ^2^ The University of Texas Health Science Center at Houston, Houston, TX 77030, USA.; ^3^ Hamon Center for Therapeutic Oncology Research, UT Southwestern Medical Center, Dallas, TX 75390, USA.; ^4^ School of Public Health, UT Southwestern Medical Center, Dallas, TX 75390, USA.; ^5^ Department of Pharmacology, UT Southwestern Medical Center, Dallas, TX 75390, USA.; ^6^ Harold C. Simmons Comprehensive Cancer Center, UT Southwestern Medical Center, Dallas, TX 75390, USA.; ^7^ Hamon Center for Regenerative Medicine, UT Southwestern Medical Center, Dallas, TX 75390, USA.

## Abstract

We identified an important oncogenic role for protocadherin 7 (PCDH7), a cell surface protein frequently overexpressed in lung adenocarcinoma and associated with poor clinical outcome. *Pcdh7* depletion reduces tumor burden and prolongs survival in *Kras^LSL-G12D^*; *Tp53^fl/fl^* mice. These findings nominate this cell surface protein as an actionable therapeutic target and highlight the therapeutic potential of PCDH7 inhibition for non–small cell lung cancer. We report the development and characterization of high-affinity anti-PCDH7 monoclonal antibodies (mAbs) that inhibit downstream mitogen-activated protein kinase (MAPK) pathway activation and suppress tumor growth in multiple mutant *KRAS*–driven models. A lead mAb (mAb7) sensitized tumors to the US Food and Drug Administration–approved MAPK kinase inhibitor trametinib and the KRAS^G12C^ inhibitor adagrasib. A humanized mAb7-IgG1 (Hu-mAb7) exhibited antibody-dependent cellular cytotoxicity and Fc-mediated immune effector killing of tumor cells in vivo. Moreover, a murinized antibody (Ms-mAb7) improved antitumor immunity in a *Kras^G12D^* syngeneic tumor model by enhancing infiltration and activation of cytotoxic immune cells. These findings provide an important advance in the clinical development of PCDH7-targeting antibodies for lung cancer treatment.

## Teaser

Antibody targeting of PCDH7 inhibits tumor growth, enhances *KRAS*-targeted therapy response, and directs tumor immunosurveillance.

## INTRODUCTION

Protocadherins (PCDHs) are transmembrane proteins and members of the cadherin superfamily. While PCDHs have well-established roles in cell adhesion, emerging data demonstrate that PCDHs are also important regulators of downstream signaling pathways (*1*–*6*). A growing body of evidence has revealed critical roles for PCDHs in human diseases, including cancer (*7*–*9*). We found that PCDH7 is frequently overexpressed in lung adenocarcinoma (LUAD) and is associated with poor clinical outcome (*7*). PCDH7 overexpression synergizes with lung cancer drivers, including mutant *KRAS*, to induce mitogen-activated protein kinase (MAPK) signaling and tumorigenesis. This work elucidated a regulatory input that promotes oncogenic RAS signaling.

Our previously established gain- and loss-of-function mouse models provided strong evidence that PCDH7 is an actionable therapeutic target (*10*). Our work demonstrated that enforced PCDH7 expression significantly accelerates *Kras^G12D^*-driven lung tumorigenesis. Conversely, inactivation of PCDH7 using CRISPR-Cas9 somatic genome editing in *Kras^LSL-G12D^*; *Tp53^fl/fl^* (KP) mice significantly reduced lung tumor development, prolonged survival, and diminished phosphoactivation of extracellular signal–regulated kinase 1/2 (ERK1/2). Moreover, we and others have uncovered important roles for PCDH7 in multiple stages of tumor biology (*7*, *10*–*12*). PCDH7 was shown to promote brain metastasis of lung cancer cells (*13*, *14*). Furthermore, PCDH7 expression was recently shown to support an immunologically “cold” tumor microenvironment (TME) in lung and pancreatic cancers (*15*, *16*). Collectively, these findings establish a critical oncogenic function for PCDH7 in vivo and highlight the therapeutic potential of PCDH7 inhibition for lung cancer.

Monoclonal antibodies (mAbs) represent an exciting development in targeted molecular cancer therapies. These molecules have an advantage over many cancer therapeutics because they are precise, recognize their target antigen with high specificity and affinity, and engage the immune system to direct antitumor immune responses (*17*–*23*). These molecules have a variety of mechanisms by which they induce tumor cell killing, both independently of the immune system (Fab-dependent function) and by directing an immune response (Fc-dependent function). Among clinically approved mAbs are those targeting immune checkpoint proteins. Clinical trials using mAbs that disrupt the PD-1 (programmed cell death protein 1)/PD-L1 (programmed death-ligand 1) immune checkpoint have yielded substantial results, with PD-1 immunotherapy approved as a first-line therapy for patients with lung cancer (*24*). However, a substantial proportion of patients with non–small cell lung cancer (NSCLC) do not exhibit durable responses to these therapies (*25*, *26*). A second class of mAbs targets tumor-associated antigens enriched on the surface of cancer cells, including EGFR (epidermal growth factor receptor) and HER2 (human epidermal growth factor receptor 2) (*27*). mAbs targeting these molecules are highly effective at neutralizing oncogenic activity and direct antitumor immune responses. However, they share disadvantages with the small-molecule inhibitors that target these proteins, including off-target toxicity, acquired resistance, and eventual relapse (*23*). Thus, the development of novel antibody-based therapies is urgently needed.

Here, we report the generation and characterization of a panel of mAbs that neutralize PCDH7. We screened antibody candidates for high affinity to PCDH7 and the ability to inhibit MAPK pathway activity and cell viability in mutant *KRAS* LUAD cell lines. mAb7 treatment phenocopied PCDH7 knockout in reducing proliferation and ERK phosphorylation and increasing apoptosis in *KRAS*-mutant xenografts. A lead candidate (mAb7) reduced tumor growth in multiple mouse models and sensitized *KRAS-*mutant xenografts to the MAPK kinase (MEK) inhibitor trametinib and the KRAS^G12C^ inhibitor adagrasib. A humanized antibody (in humanized mice) and a murinized antibody (in wild-type mice) diminished tumor growth in an Fc-dependent manner, selectively enhancing antibody-dependent cellular cytotoxicity (ADCC) and granzyme B (GrzB) production. Collectively, these findings demonstrate that PCDH7 blockade is a promising therapeutic strategy for KRAS-driven LUAD and potentially other malignancies with high PCDH7 expression.

## RESULTS

### Generation and functional characterization of PCDH7-targeting mAbs

**Fig. 1.**
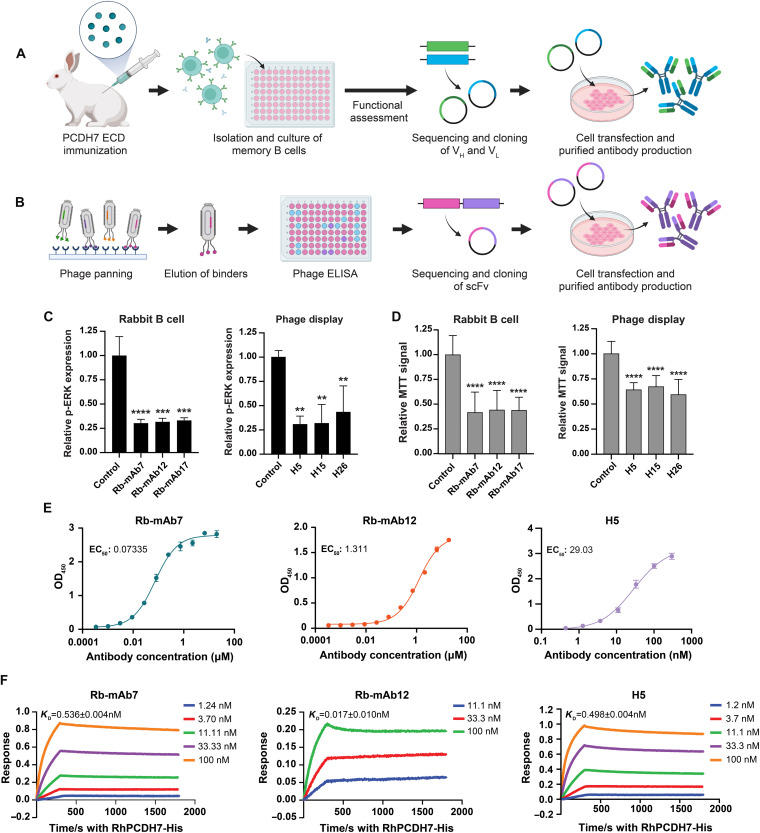
Generation and functional characterization of PCDH7-targeting mAbs. (**A** and **B**) Overview of rabbit B cell (A) and phage display (B) antibody generation [created in BioRender. O’Donnell, K. (2026), https://biorender.com/1zcapxm]. (**C**) LiCOR ICW analysis of p-ERK expression in cells treated with top three candidates from each antibody panel. The target signal was normalized to CellTag 700 and reported relative to the control antibody. (**D**) MTT assay data showing cell viability in top three candidates from rabbit B cell and phage display antibody groups. The MTT signal was reported relative to control antibody values. For all ICW and MTT assays, statistics were determined by a one-way analysis of variance (ANOVA) with Dunnett’s multiple comparison test. ****P < 0.0001, ***P 0.0001, and **P < 0.005. Error bars indicate SD and *n* = 3. Experiments were repeated twice for confirmation. (**E**) PCDH7 mAb binding affinity determination with antibody titration ELISA. OD_450_, optical density at 450 nm; EC_50_, median effective concentration. (**F**) PCDH7 mAb kinetic binding measurements using BLI-based Octet detection. Detection used protein A biosensors and PCDH7-ECD (6HIS tag) as the analyte at different concentrations.

To assess the efficacy of targeting PCDH7 in NSCLC, we generated a panel of PCDH7-neutralizing mAbs using two previously described approaches (Fig. 1A) (*28*, *29*). First, New Zealand rabbits were immunized with the purified PCDH7 extracellular domain (ECD) (fig. S1A). Rabbits with the highest serum titers were selected for antibody-producing B cell isolation and single B cell culture. One week after injection of the final boost, 400 plasma B cells were isolated from peripheral blood mononuclear cells (PBMCs) by fluorescence-activated cell sorting (FACS) and grown as single-cell clones. Supernatants from these clones were screened by enzyme-linked immunosorbent assay (ELISA) for PCDH7 binding, and 151 candidates were identified as high-affinity binders. A subset of these strong binders, identified through further functional assays described below, was then used to clone antibody sequences. Reverse transcription polymerase chain reaction (RT-PCR) and nested PCR reactions were performed following the isolation of individual plasma B cells to amplify the DNA sequences of individual antibody heavy and light chains. The sequences were then cloned into mammalian expression vectors for transient transfection into human embryonic kidney (HEK) 293 cells, and the antibodies were purified.

In a second approach, we developed human mAb candidates using single-chain variable fragment (scFv) phage display technology (Fig. 1B). A high-diversity (5 × 10^11^) scFv phage display human antibody library was panned for PCDH7-specific antibodies, and three rounds of solid-phase panning were performed to select desirable antigen-binding hits. A total of 387 phages were then tested by ELISA for high-affinity binding to PCDH7, yielding 30 scFv candidates. The antibody genes encoding these candidates were sequenced and used to generate full-length antibody molecules by cloning into an expression vector for human immunoglobulin G1 (IgG1) production in HEK293 cells.

The efficacy of the B cell and phage display antibody candidates was evaluated using a series of functional assays. Given our previous findings demonstrating that PCDH7 synergizes with mutant *KRAS* to induce MAPK signaling and tumorigenesis in vitro and in vivo, we focused on identifying candidates that reduce phospho-ERK (p-ERK) activation, a readout of MAPK activity, and proliferation in human lung cancer cells. H1944 cells were selected because they harbor a *KRAS* mutation and express high levels of endogenous PCDH7. Using p-ERK in-cell Western (ICW) and 3-(4,5-dimethylthiazol-2-yl)-2,5-diphenyltetrazolium bromide (MTT) cell viability assays, we screened 151 rabbit plasma B cell supernatants. Following screening, we selected 19 B cell antibodies for purification and further analyses along with 23 phage display antibodies. Purified B cell antibodies were produced using variable regions of heavy- and light-chain sequences from the selected supernatants. Of these, 11 of 19 rabbit B cell mAbs and 14 of 23 phage display antibodies significantly reduced ERK phosphorylation relative to the isotype control (Fig. 1C and fig. S1, B to D). These mAbs also significantly reduced cell viability (Fig. 1D and fig. S1, E and F).

Next, we measured the binding affinities and kinetics of the selected purified mAbs. Antibody titration ELISAs were performed to assess each antibody’s binding affinity for the ECD of PCDH7 (Fig. 1E). Antibody kinetic binding measurements were performed using biolayer interferometry (BLI)–based Octet detection (Fig. 1F). ForteBio’s data analysis software was used to determine the association rate (*k*_on_) and dissociation rate (*k*_off_), and *K*_D_ (dissociation constant) was calculated as the ratio of *k*_off_/*k*_on._ The binding affinities of the PCDH7 mAbs are shown in table S1. On the basis of the high affinities for PCDH7 and strong functional data showing reduced p-ERK activation and viability in LUAD cells, we selected three candidates (Rb-mAb7, Rb-mAb12, and H5) for further characterization and in vivo testing.

### PCDH7-neutralizing antibodies reduce the tumor growth of *KRAS*-mutant xenografts

**Fig. 2.**
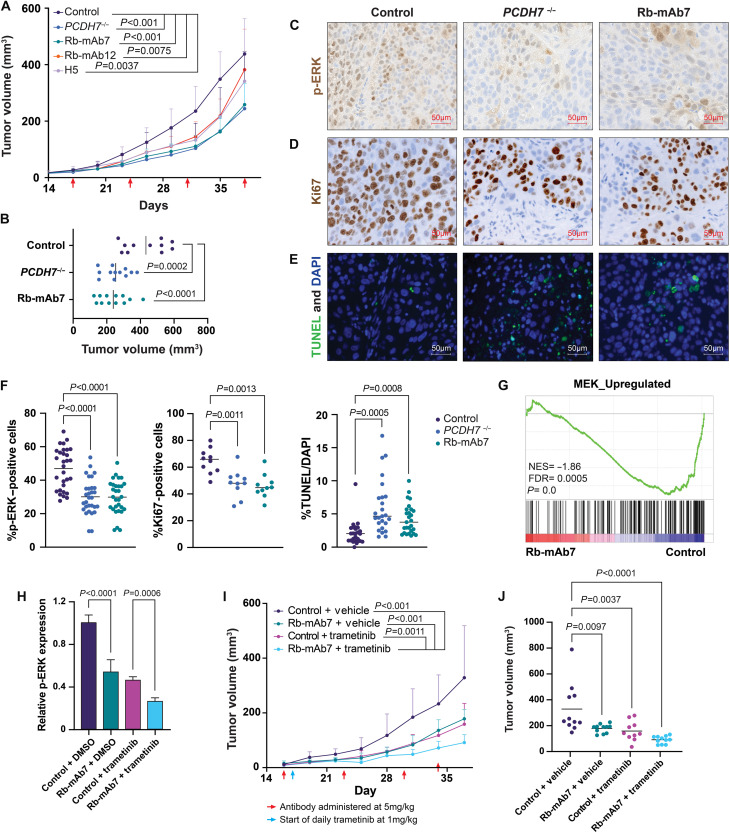
PCDH7-neutralizing antibodies reduce the tumor growth of *KRAS*-mutant xenografts alone and in combination with the MEK inhibitor trametinib. (**A**) Tumor volumes of H1944 or H1944 *PCDH7^−/−^* cells treated weekly with the designated antibodies (10 mg/kg) in immunocompromised NSG mice (*n* = 11 or 12) until the end point at day 38. Statistics were generated by generalized linear mixed models (GLMMs). (**B**) Final tumor volumes for control antibody, *PCDH7^−/−^*, and Rb-mAb7–treated mice from (A). Error bars indicate SD, and statistics were generated by a one-way ANOVA with Dunnett’s multiple comparison test. (**C** to **E**) IHC staining for p-ERK (C) and Ki67 (D) expression and TUNEL staining (E) performed on tumor sections from (A). (**F**) Quantitation of p-ERK, Ki67, and TUNEL staining shown in (C) to (E). For p-ERK, *n* = 3 sections with 10 fields per section. For Ki67, *n* = 2 sections with 5 fields per section. For TUNEL, *n* = 3 sections with 10 fields per section. Statistics were generated by a one-way ANOVA with Dunnett’s multiple comparison test. (**G**) GSEA enrichment plot for MEK_UP identified as down-regulated in Rb-mAb7–treated samples following RNA-seq analysis (*n* = 4 tumors per group). NES, normalized enrichment score. (**H**) ICW data showing p-ERK expression in cells following indicated treatment, reported relative to control + DMSO values. Error bars indicate SD and *n* = 3. Experiments were repeated twice for confirmation. (**I**) Tumor volumes of mice harboring H1944 xenografts treated weekly with Rb-mAb7 (5 mg/kg) with or without daily dosing of trametinib (1 mg/kg) until the end point at day 37 (*n* = 10 or 11). Statistics were generated by GLMMs. (**J**) final tumor volumes for mice from (I). Statistics were generated by a one-way ANOVA with Dunnett’s test for multiple comparisons.

The therapeutic efficacy of the top three candidates was assessed using antibody treatment in immunocompromised NOD/SCID Il2rγ^**−/−**^ (NSG) mice harboring *KRAS*-mutant LUAD xenografts. Mice were treated with either IgG control, Rb-mAb7, Rb-mAb12, or H5 antibodies via weekly intraperitoneal injection (10 mg/kg) after forming palpable tumors. An additional group of control mice was transplanted with lung cancer cells depleted of *PCDH7* with CRISPR-Cas9 editing (H1944 *PCDH7^−/−^*). All three PCDH7-neutralizing mAbs significantly reduced tumor growth relative to the isotype control antibody (Fig. 2A). Tumor growth of the Rb-mAb7 treatment group was nearly identical to *PCDH*7 knockout tumors (Fig. 2, A and B). Moreover, treatment with Rb-mAb7 phenocopied *PCDH7* knockout, resulting in similar effects on cell proliferation, apoptosis, and MAPK activation, as assessed by Ki67 immunohistochemistry (IHC), terminal deoxynucleotidyl transferase–mediated deoxyuridine triphosphate nick end labeling (TUNEL) staining, and p-ERK IHC, respectively (Fig. 2, C to F). Following these results, we focused on mAb7 as a lead candidate for further characterization and in vivo testing.

RNA sequencing (RNA-seq) was performed in tumors to assess global gene expression changes in response to Rb-mAb7 treatment and *PCDH7* genetic depletion using CRISPR-Cas9. Gene set enrichment analysis (GSEA) identified significant down-regulation of MAPK signaling in Rb-mAb7–treated tumors, consistent with our previous findings (Fig. 2G). Several oncogenic signaling pathways were down-regulated in mAb7-treated tumors compared to IgG-treated control tumors (table S2). GSEA and Ingenuity Pathway Analysis further revealed significant alterations in cellular assembly and organization and cell morphology (fig. S2A), consistent with a recent study demonstrating a role for PCDH7 in regulating myosin activity and cell motility (*30*).

Antibody treatments were well tolerated without notable toxicity, as demonstrated by consistent body weights across groups (fig. S2, B and C). Serum analyses confirmed that all antibody candidates remained stable in blood serum at day 20 and at the experimental end point (day 38) (fig. S2D). Dosage studies were also performed with Rb-mAb7 (1, 5, or 10 mg/kg). Rb-mAb7 significantly reduced the tumor growth of human NSCLC xenografts at both 5 and 10 mg/kg (fig. S2, E and F).

Our observation that PCDH7 activates the MAPK pathway in cooperation with mutant *KRAS* suggests that PCDH7 may modulate the response of *KRAS*-mutant NSCLC cells to clinically approved MAPK inhibitors. Consistent with this, PCDH7 expression promotes BRAF inhibitor resistance in melanoma cells (*31*). We therefore tested the ability of Rb-mAb7 to synergize with the US Food and Drug Administration–approved MEK inhibitor trametinib in vitro and in vivo. Using ICWs, we observed a significant reduction in ERK phosphorylation in LUAD cells treated with trametinib and Rb-mAb7 compared to cells treated with trametinib alone or an IgG control antibody (Fig. 2H). To assess the combinatorial effects in vivo, NSG mice harboring NSCLC xenografts were treated with an IgG control or Rb-mAb7 antibody via weekly intraperitoneal injection (5 mg/kg) and either vehicle or trametinib (1 mg/kg) via daily oral gavage. As expected, trametinib and Rb-mAb7 alone significantly reduced tumor burden relative to control treatments. Moreover, the combination of Rb-mAb7 and trametinib significantly reduced tumor burden compared to either monotherapy, indicating that Rb-mAb7 sensitizes tumors to MEK inhibition (Fig. 2, I and J).

### Binding specificity of Rb-mAb7

**Fig. 3.**
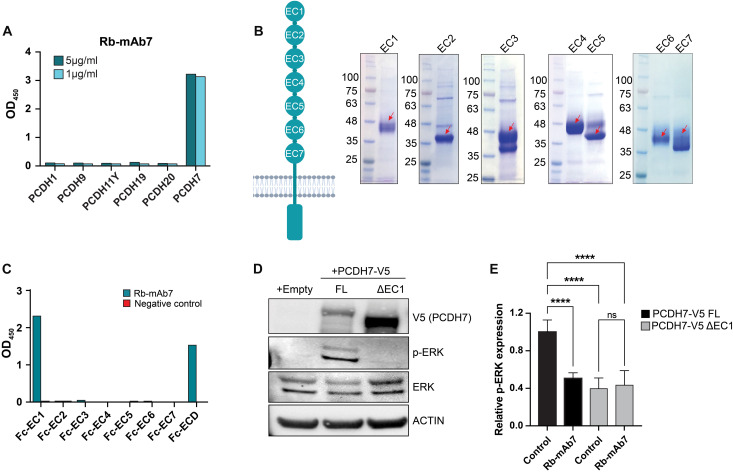
Binding specificity of Rb-mAb7. (**A**) Evaluation of PCDH7 antibody cross-reactivity to δ1 PCDH family members by ELISA. (**B**) Left: Schematic representation of PCDH7 with seven EC repeats (EC1 to EC7) [created in BioRender. O’Donnell, K. (2026), https://biorender.com/pm9x209]. Right: Coomassie gel of purified PCDH7 EC domain peptides. (**C**) Rb-mAb7 binding affinity determination for PCDH7 EC domains 1 to 7 with antibody titration ELISA. (**D**) Western blot analysis of HBEC-*shp53; KRAS^G12V^* cells expressing an empty vector, PCDH7-V5 FL, or PCDH7-V5 ΔC1. Actin served as a loading control. Images from a single experiment are shown and are representative of three independent experiments. (**E**) ICW data showing p-ERK expression in HBEC- *shp53; KRAS^G12V^*; PCDH7-V5 and HBEC-*shp53; KRAS^G12V^*; PCDH7ΔC1-V5 cells treated with control or Rb-mAb7. Error bars indicate SD and *n* = 3. Experiments were repeated twice for confirmation. ns, not significant. ****P < 0.0001 by one-way ANOVA.

δ-PCDHs are a family of nonclustered cell surface proteins characterized by cadherin-like ECDs composed of six or seven extracellular cadherin (EC) repeats and a unique cytoplasmic domain (*32*). PCDH7 is a member of the δ1 PCDH family and shares 67.4% sequence similarity with PCDH1, its most closely related family member (fig. S3A). It was therefore important to establish the target specificity of the antibody candidates to avoid potential off-target binding and toxicity. Using ELISAs, we assessed antibody binding affinities for all four δ1 PCDH family members, PCDH1, PCDH9, PCDH11, and PCDH7; the structurally similar PCDH10; and the δ2 PCDH family member PCDH19. These experiments revealed that Rb-mAb7, Rb-mAb12, and H5 selectively bind PCDH7 and do not cross-react with other human PCDH family members (Fig. 3A and fig. S3B). Rb-mAb7 and H5 but not mAb12 exhibit significant binding to murine PCDH7 (fig. S3, C to E), suggesting that mAb7 or H5 may neutralize PCDH7 function in mouse models.

Like other δ1 PCDH family members, PCDH7 has a characteristic extracellular region composed of seven EC repeat domains (*9*). To determine which EC domain Rb-mAb7 binds, we generated individual EC domains or the full-length ECD (with a human Fc tag at the C terminus), and protein purity was assessed by SDS–polyacrylamide gel electrophoresis (Fig. 3B). Rb-mAb7 exhibited a strong affinity for EC1, indicating highly specific binding to this region of the ECD (Fig. 3C).

To assess the specificity and functional consequence of mAb7 binding to EC1, we generated two V5-tagged PCDH7 constructs, one expressing the full-length protein (PCDH7-V5 FL) and another with deletion of the EC1 domain (PCDH7-V5 ΔC1). Consistent with our previous findings, enforced expression of PCDH7-V5 FL potently induced ERK phosphorylation in HBEC-shp53*-KRAS^G12V^* cells (where HBEC refers to human bronchial epithelial cell) relative to cells transduced with an empty vector (*7*). In contrast, enforced expression of PCDH7-V5 ΔC1 did not induce ERK phosphorylation (Fig. 3D). Moreover, ICW experiments demonstrated that p-ERK levels were suppressed in PCDH7-V5 FL–expressing cells, but not in PCDH7-V5 ΔC1–expressing cells, upon treatment with Rb-mAb7 compared to a control antibody (Fig. 3E). Together, these data revealed that PCDH7 EC1, containing a high-affinity epitope recognized by Rb-mAb7, is essential for promoting downstream MAPK signaling.

### Humanization and functional characterization of Hu-mAb7

**Fig. 4.**
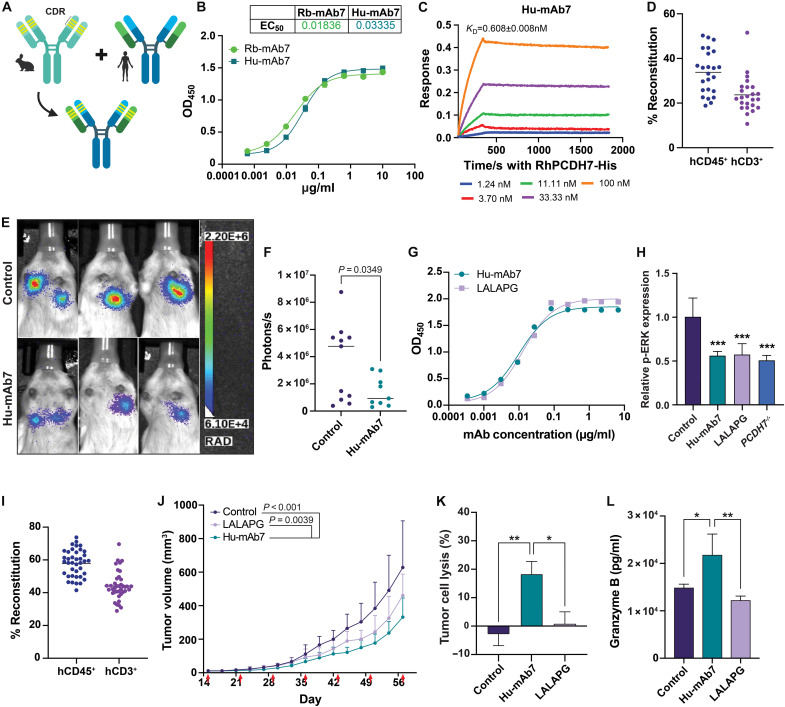
Humanization and functional characterization of Hu-mAb7. (**A**) Overview of rabbit mAb humanization [created in BioRender. O’Donnell, K. (2026), https://biorender.com/fgqoyv6]. (**B**) Binding affinity of humanized mAb7 using antibody titration ELISA. (**C**) Hu-mAb7 kinetic binding measurements using BLI-based Octet detection. (**D**) Flow cytometry data confirming hCD45^+^ and hCD3^+^ cells in peripheral blood of humanized NSG mice 12 weeks after CD34^+^ cell engraftment. (**E**) Representative bioluminescence imaging of H1944 orthotopic lung tumors in humanized NSG mice, 4 weeks postinoculation. Mice were treated weekly with control or Hu-mAb7 (10 mg/kg) antibodies and imaged following luciferin injection (*n* = 9 to 11). (**F**) Quantification of orthotopic tumor luciferase signal from (E). Analysis was performed by an unpaired *t* test. (**G**) Binding affinity of humanized mAb7-LALAPG using antibody titration ELISA. (**H**) ICW data for p-ERK in control-, Hu-mAb7–, or LALAPG-treated cells or *PCDH7^−/−^* cells. Error bars indicate SD and *n* = 3. Experiments repeated twice for confirmation. (**I**) Flow cytometry data confirming hCD45^+^ and hCD3^+^ cells in the peripheral blood of humanized NSG-SGM3 mice 12 weeks after CD34^+^ cell engraftment. (**J**) Tumor volumes of humanized mice from (I) harboring H1944 xenografts treated with the designated antibodies (10 mg/kg) (*n* = 9 to 11) until day 57. Red arrows denote antibody treatments. Statistics generated by GLMMs. (**K**) ADCC data representing tumor cell lysis when cocultured with PBMCs and treated with indicated antibodies. Tumor lysis (%) was calculated by (cell index of control group − cell index of treatment group)/(cell index of control group) × 100. The *y* axis shows the average of three independent assays (*n =* 3), and error bars indicate SD. Analysis was performed by an unpaired *t* test. *P < 0.05 and **P < 0.01. (**L**) GrzB ELISA for NK-92 MI cells cocultured for 24 hours with PC9 cells overexpressing PCDH7 and treated with the indicated antibodies. Statistics generated by a one-way ANOVA. Error bars indicate SD and *n* = 3. Experiments were repeated three times for confirmation. ***P = 0.0001 by one-way ANOVA.

For potential therapeutic development, we humanized Rb-mAb7 using a previously described complementarity-derived region grafting strategy (Fig. 4A) (*28*). Briefly, the mAb7 complementarity-derived regions were grafted into a human germline framework using a human antibody sequence database. The humanized V_H_ (variable region of immunoglobulin heavy chain) and V_L_ (variable region of immunoglobulin light chain) fragments were cloned into an expression vector for full-length IgG1 expression and purification. Next, we assessed the binding affinity of the humanized antibodies by ELISA and Octet detection. We selected PCDH7(Hu)H1K1 (Hu-mAb7) for further characterization because of its strong binding affinity for the PCDH7 ECD and its similarity to Rb-mAb7 in binding affinity (Fig. 4, B and C, and fig. S4A).

Treatment with either Rb-mAb7 or Hu-mAb7 significantly reduced ERK phosphorylation and cell viability in human H1944 lung cancer cells, as measured by ICW and MTT assays, respectively (fig. S4, B and C). This effect persisted in vivo in tumor-bearing NSG mice treated with IgG control, Rb-mAb7, or Hu-mAb7 antibodies (10 mg/kg). Mice treated with Rb-mAb7 or Hu-mAb7 demonstrated significantly reduced tumor growth compared to mice treated with the IgG control, with growth curves that were indistinguishable between the two mAb7 groups (fig. S4, D and E). These results indicated that Hu-mAb7 maintained the PCDH7 blocking activity of the parental rabbit mAb7.

On-target specificity of Hu-mAb7 was established through functional assays in LUAD cells with high endogenous PCDH7 and in PCDH7 knockout cells (fig. S4F). Following confirmation of reduced cell growth in PCDH7 knockout H1944 cells (fig. S4G), the response to Hu-mAb7 was assessed by ICW and MTT assays. These results demonstrated that no additional reduction of ERK phosphorylation or cell viability occurred in PCDH7 knockout cells with Hu-mAb7 treatment (fig. S4, H and I). Additional testing in six *KRAS*-mutant LUAD cell lines was performed to determine the extent to which antibody efficacy depends on PCDH7 expression. We selected three *KRAS*-mutant cell lines with medium to high levels of PCDH7 expression (H1944, HOP-62, and DFCI024) and three with low baseline PCDH7 expression (H2030, HCC515, and H2887) (fig. S5A). Hu-mAb7 treatment significantly reduced p-ERK activity and cell proliferation compared to control IgG in the three PCDH7 medium- to high-expressing cell lines but not in the PCDH7 low-expressing cell lines, indicating that there is a threshold for Hu-mAb7 efficacy that depends on PCDH7 expression (fig. S5, B and C).

In addition, Hu-mAb7 sensitized *KRAS^G12C^*-mutant cells to the recently approved KRAS inhibitor adagrasib (MRTX849), as demonstrated by the decreased cell viability of DFCI024 cells, a *KRAS^G12C^*-mutant NSCLC cell line, treated with both Hu-mAb7 and adagrasib (fig. S6A). In vivo, monotherapy with Hu-mAb7 or adagrasib comparably reduced the growth of DFCI024 xenograft tumors, while combination therapy outperformed either treatment alone (fig. S6B). Furthermore, to assess the efficacy of Hu-mAb7 in adagrasib-resistant tumors, we established cell lines from DFCI024 xenografts that did not respond to adagrasib treatment in vivo (fig. S6C). We found that the adagrasib-resistant cell lines (DFCI024 AR1-3) expressed higher PCDH7 mRNA and protein levels compared to the parental DFCI024 cells (fig. S6, D and E). Treatment of the resistant cells with Hu-mAb7 decreased cell viability in MTT assays (fig. S6F), and consistent with this result, treatment of nude mice harboring DFCI024 AR xenograft tumors with Hu-mAb7 significantly reduced tumor growth (fig. S6G).

Humanization of mAb7 enabled antibody testing in the presence of a humanized immune system. We therefore generated humanized mice using a previously established method (*33*, *34*). Briefly, we ablated bone marrow cells of NSG mice with sublethal irradiation and then reconstituted the immune system by intravenous engraftment of CD34^+^ hematopoietic stem cells isolated from fresh human umbilical cord blood. Twelve weeks after engraftment, we validated reconstitution by FACS analysis of peripheral blood, defining mice with >20% human CD45^+^ (hCD45^+^) immune cells in circulation as successfully humanized (Fig. 4D). Humanized mice were orthotopically transplanted with H1944 cells stably expressing *Renilla* luciferase and treated with an IgG control or Hu-mAb7 (10 mg/kg) weekly via intraperitoneal injection. Whole-body bioluminescence imaging was performed weekly to monitor tumor growth. At final imaging (4 weeks postinjection), mice treated with Hu-mAb7 had significantly reduced tumor burden by whole-body bioluminescence imaging compared to IgG control–treated mice (Fig. 4, E and F).

To evaluate the ability of Hu-mAb7 to direct an antitumor immune response in an Fc-dependent manner, we generated a mutant Hu-mAb7-LALAPG (LALAPG) antibody, which does not engage with Fc receptors (*35*, *36*). These antibodies are engineered with L234A/L235A/P329G mutations, rendering them effector-silent because of impaired interactions with Fcγ receptors and complement component 1q (*36*). We confirmed that Hu-mAb7 and LALAPG have similar binding affinity for PCDH7 and behave identically in reducing ERK phosphorylation and cell viability in the absence of immune cells in vitro (Fig. 4, G and H, and fig. S7A), indicating that PCDH7-neutralizing activity persists in the LALAPG mutant. For in vivo testing, we humanized NSG-SGM3 mice, which have higher rates of human immune cell engraftment than NSG mice (*37*). These mice exhibited an average reconstitution of 60% hCD45^+^ cells in peripheral blood, with all mice having more than 40% hCD45^+^ cells (Fig. 4I). Following confirmation of humanization, H1944 cells were transplanted subcutaneously into the flanks of humanized mice. After the formation of palpable tumors (day 15), weekly treatments with IgG control, Hu-mAb7, or LALAPG antibodies (10 mg/kg) were initiated. Both Hu-mAb7 and LALAPG antibody–treated mice showed significantly reduced tumor growth compared to IgG control–treated mice (Fig. 4J). Furthermore, growth of Hu-mAb7–treated tumors was significantly reduced compared to the Hu-mAb7-LALAPG–treated tumors (Fig. 4J). These data demonstrate that Hu-mAb7 can reduce tumor growth both by inhibiting PCDH7 signaling and by directing an antitumor immune response in an Fc-dependent manner.

One critical immune-associated function of antibodies is their ability to engage and direct ADCC (*17*). To evaluate Hu-mAb7’s ability to perform this function, we implemented ADCC coculture assays using PBMCs and human NSCLC cells expressing PCDH7-V5. In this assay, treatment with Hu-mAb7 induced lysis of ~20% of PCDH7-V5–expressing NSCLC cells, while no lysis was detected following IgG or LALAPG treatment (Fig. 4K). To further investigate the immune-stimulatory effect of Hu-mAb7 treatment, we performed coculture experiments with human NSCLC cells and natural killer (NK) cells to assess GrzB production. GrzB is a cytotoxic serine protease secreted by NK cells and cytotoxic CD8^+^ T cells to induce apoptosis in target cells (*38*). Using an ELISA, we observed a significant increase in GrzB release from NK cells treated with Hu-mAb7 compared with control IgG or LALAPG antibodies (Fig. 4L and fig. S7B). Similar results were observed upon coculture of activated Jurkat T cells with human NSCLC cells treated with the Hu-mAb7 antibody (fig. S7C). Collectively, these data demonstrate that Hu-mAb7 can mediate Fc-dependent immune effector function.

### Chimeric mAb7 enhances cytotoxic immunity in a *Kras^G12D^*-mutant syngeneic tumor model

**Fig. 5.**
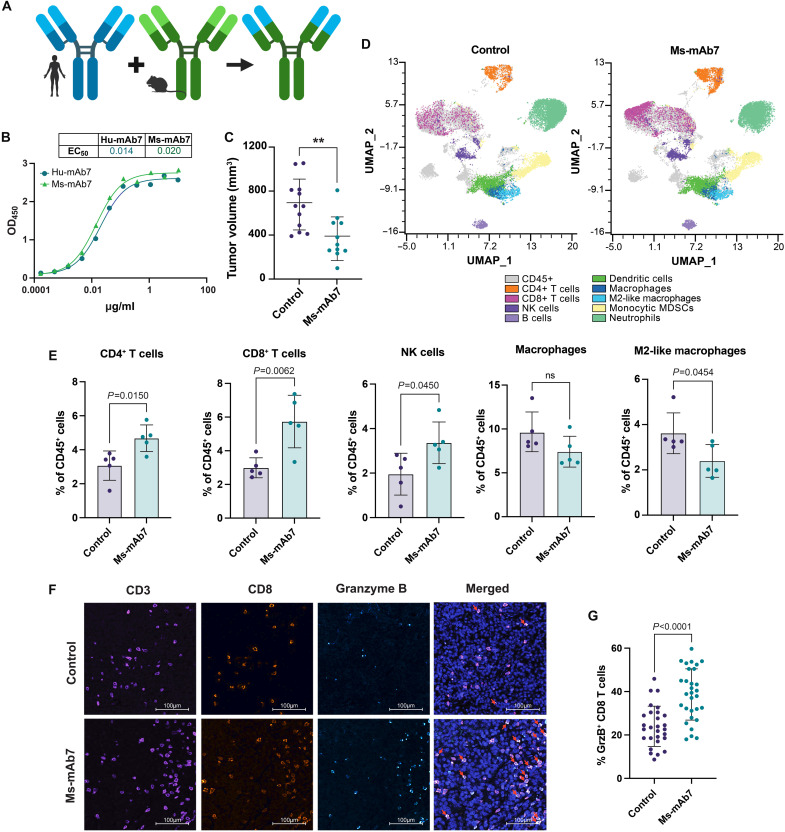
Murinized mAb7 reduces tumor burden and promotes tumor infiltration by cytotoxic immune cells. (**A**) Overview of Hu-mAb7 murinization [created in BioRender. O’Donnell, K. (2026), https://biorender.com/gp499mj]. (**B**) Binding affinity of murinized mAb7 using antibody titration ELISA. (**C**) Final volumes of KP67-1 syngeneic tumors in C57BL/6 mice treated with the designated antibody (20 mg/kg) every 3 days (*n* = 11 or 12). Statistics were generated by an unpaired *t* test (**P = 0.0035). (**D**) Uniform manifold approximation and projection (UMAP) analysis of TILs colored by cell types. (**E**) Quantification of TILs (expressed as a percentage of CD45^+^ cells) identified by mass CyTOF from mice in (C) (*n* = 5 per treatment). The following markers were used for each CD45^+^ cell type: CD4^+^ T cells (CD3^+^CD4^+^), CD8^+^ T cells (CD3^+^CD8^+^), NK cells (CD3^−^NK1-1^+^), B cells (CD19^+^B220^+^), dendritic cells (CD11b^+^CD11c^+^IA/IE^+^), macrophages (CD11b^+^F4/80^+^), M2-like macrophages (CD11b^+^F4/80^+^ CD206^+^), monocytic myeloid-derived suppressor cells (MDSCs) (CD11b^+^Ly-6C^+^Ly-6G^−^), and neutrophils (CD11b^+^Ly-6G^+^IA/IE^−^). *P* values were generated by an unpaired *t* test. (**F**) Multiplexed IHC-F was performed on tumors from (C), with 10× representative images shown. Scale bars, 100 μm. Red arrows indicate GrzB^+^ CD8^+^ T cells. (**G**) Percentage of GrzB^+^ CD8^+^ T cells relative to total CD8^+^ T cells. For each treatment group, 10 fields were quantified per tumor from three mice. Statistics were generated by an unpaired *t* test (*P* < 0.0001).

To more fully assess the ability of mAb7 to direct immune surveillance, we engineered a murinized antibody (Ms-mAb7) containing humanized mAb7 variable sequences fused with a mouse IgG2a constant region (Fig. 5A). As expected, Ms-mAb7 binds PCDH7 with similar affinity to Hu-mAb7 in ELISAs (Fig. 5B). We assessed the functional consequences of Ms-mAb7 treatment in KP67-1 cells, a cell line with high PCDH7 expression (fig. S8A) derived from the autochthonous KP mouse model (*39*). Treatment with Ms-mAb7 significantly reduced ERK phosphorylation and cell viability in a Fab-dependent manner (fig. S8B). Next, we assessed the Fc-dependent antitumor efficacy of Ms-mAb7 in an intact immune system by transplanting KP67-1 cells into the flanks of wild-type C57BL/6 mice. Ms-mAb7 or IgG control antibodies were administered every 3 days. Ms-mAb7 significantly reduced tumor growth compared to control-treated tumors (Fig. 5C). The target specificity of Ms-mAb7 was confirmed by testing three *Kras*-mutant murine LUAD cell lines, two with high endogenous PCDH7 expression (KP and KdP67-1) and one with little to no PCDH7 expression (CMT167) (fig. S8C). ICW and MTT assays revealed that the murinized Ms-mAb7 had no functional effect in CMT167 cells (fig. S8, D and E), further demonstrating that antibody efficacy depends on PCDH7 expression.

To quantitatively evaluate TME changes, syngeneic tumors were harvested at the end point and processed for flow mass cytometry by time of flight (mass CyTOF). Labeled cells were filtered using previously described gating strategies to identify singlets (*40*), and live CD45^+^ immune cells were identified (CD45^+^, cisplatin^−^), then further gated by cell type (fig. S8F). Uniform manifold approximation and projection (UMAP) analysis of tumor-infiltrating lymphocytes (TILs) was performed for dimension reduction and annotated by cell type (Fig. 5D). Quantification of changes in specific cell populations revealed significant increases in both helper (CD4^+^) and cytotoxic (CD8^+^) T cell infiltration, as well as in NK cell infiltration, following Ms-mAb7 treatment (Fig. 5E and fig. S8G). In addition, the percentage of M2-like macrophages, an immunosuppressive cell population, was reduced in Ms-mAb7–treated tumors. These changes in TIL composition, namely the increase in cytotoxically active cells (CD8^+^ T cells and NK cells) and the decrease in inhibitory M2-like macrophages, suggest a shift toward a more tumor-suppressive TME upon Ms-mAb7 treatment. To further evaluate the activity of CD8^+^ T cells following Ms-mAb7 treatment, we performed multiplex fluorescent IHC (IHC-F) staining to assess GrzB production (Fig. 5F). This confirmed elevated infiltration of CD8^+^ T cells and revealed an increased percentage of GrzB^+^ CD8^+^ T cells in Ms-mAb7–treated tumors (Fig. 5G and fig. S8H).

## DISCUSSION

Despite continued efforts toward the development of targeted therapies for lung cancer, it remains the deadliest cancer type worldwide. For patients with advanced NSCLC, small-molecule inhibitors are often used as first-line therapy because of their initial efficacy. However, these often target essential and ubiquitously expressed proteins, leading to toxicity. Furthermore, currently available monotherapies often result in disease relapse. mAbs for cancer therapy have risen in prominence because of their ease of clinical development, high target specificity, and ability to neutralize oncogenic protein activity (*41*). These features, along with their ability to engage the antitumor immune response in an Fc-dependent manner, make them highly effective as cancer therapies. Notably, most US Food and Drug Administration–approved mAb therapies target only a small number of molecules, most of which are immune checkpoint proteins. Thus, identifying and characterizing novel cell surface proteins up-regulated in tumor cells should expand the repertoire of antibody-based therapeutics.

The overexpression of PCDH7 on the cell surface of lung cancer cells, along with its important role in modulating the MAPK pathway, makes it an attractive target for novel anticancer mAb therapies. We previously described the role of PCDH7 in lung tumor initiation and progression, and other studies have demonstrated a role for PCDH7 in promoting brain metastasis of lung and breast cancer cells (*7*, *10*, *14*). These findings illustrate the importance of this protein at multiple stages of tumor biology and provide a rationale for therapeutic targeting of this cell surface protein. We demonstrated that genetic depletion of *Pcdh7* in the KP mouse model of LUAD significantly abrogated lung tumor development, indicating that PCDH7 neutralization is sufficient to reduce tumor burden. These findings, along with the observation that *Pcdh7^−/−^* mice are viable, led us to hypothesize that PCDH7 may be a viable therapeutic target.

This study tested this hypothesis by generating a panel of mAbs targeting PCDH7. Functional screening was performed to identify antibody candidates with the following characteristics: (i) high specificity and affinity for the PCDH7 ECD, (ii) the ability to reduce MAPK pathway activation and cell viability in multiple LUAD cell lines, (iii) the ability to inhibit tumor growth in multiple LUAD xenograft models, and (iv) the ability to engage the antitumor immune response in an Fc-dependent manner. Using these criteria, we identified a PCDH7-targeting antibody, mAb7, as a lead candidate for potential therapeutic use. We determined the binding specificity of Rb-mAb7 for PCDH7, showing that this antibody does not bind other members of the δ-1 PCDH family that share high sequence similarity and identity with PCDH7, or other structurally similar cadherins. Furthermore, we identified the EC1 domain of PCDH7 as the critical binding epitope of Rb-mAb7. Using both in vitro and in vivo models, we assessed the preclinical efficacy of mAb7. These functional assays demonstrated that antibody-mediated blockade of PCDH7 was as effective as genetic depletion in reducing tumor growth in mutant *KRAS-*driven LUADs. Furthermore, mAb7 administration alone was as effective as clinically approved inhibitors targeting the MAPK pathway in vitro and in vivo. Combined anti-PCDH7 and MEK inhibition further increased efficacy, as did the combination of anti-PCDH7 and KRAS^G12C^ inhibition, suggesting that PCDH7 blockade in combination with these targeted therapies may represent a promising strategy for KRAS-driven NSCLC. Furthermore, these data indicate that PCDH7 antibodies may be used in the setting of KRAS inhibitor resistance, an important unmet need for patients with lung cancer (*42*).

Upon humanization of mAb7, we validated its high binding affinity for PCDH7 and investigated the in vivo efficacy. We used humanized mice to assess the ability of Hu-mAb7 to synergize with functional human immune cells in vivo and, using whole-body bioluminescence imaging, found that it reduced orthotopic lung tumor burden. By engineering an “effector-silent” LALAPG-mutant Hu-mAb7, we demonstrated that Hu-mAb7 can suppress tumor growth both by directly inhibiting PCDH7 activity and by directing antitumor immune cell activity. This effect was in part due to ADCC, as evidenced by increased tumor cell lysis in PBMC coculture assays treated with Hu-mAb7. We acknowledge that an off-target immune mechanism cannot be ruled out in the humanized setting. Last, by engineering a murinized mAb7, we identified the TIL populations critical to mAb7-mediated tumor suppression. Mass CyTOF analysis revealed increased infiltration of CD4^+^ T cells and two cytotoxic populations, CD8^+^ T cells and NK cells, in Ms-mAb7–treated tumors. Furthermore, IHC-F staining confirmed elevated infiltration of CD8^+^ T cells and increased GrzB production in Ms-mAb7–treated tumors. Although direct immune cell depletion studies are necessary to determine a causative role for these immune cell populations, our results indicate that mAb7 treatment promotes antitumor immunosurveillance.

**Fig. 6.**
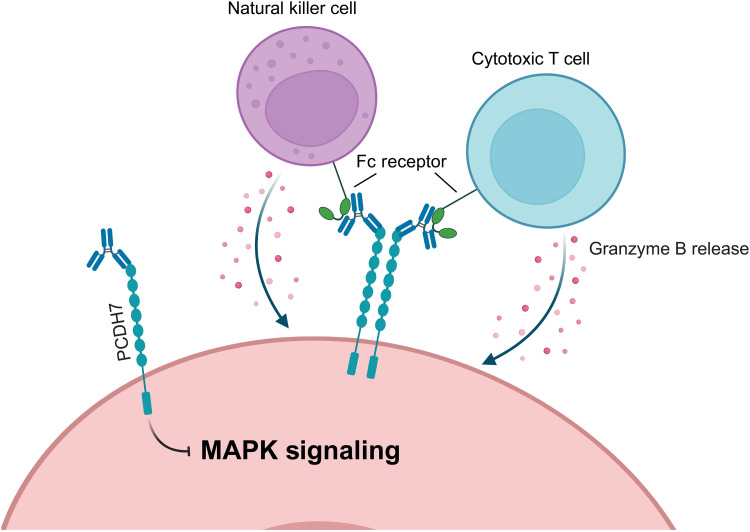
Model of PCDH7 mAb7–mediated tumor inhibition. Anti-PCDH7 mAb7 inhibits *KRAS-*driven lung tumorigenesis by inhibiting PCDH7-mediated downstream MAPK pathway activation and by triggering antibody-dependent cellular cytotoxicity (ADCC) [created in BioRender. O’Donnell, K. (2026), https://biorender.com/0xn49ht].

Collectively, this work provides strong support for the clinical development of a PCDH7-targeting mAb. Hu-mAb7 exhibits high specificity and affinity for PCDH7 and can neutralize PCDH7-driven MAPK signaling while also engaging cytotoxic TILs (Fig. 6). *Pcdh7^−/−^* mice are viable and display no overt developmental abnormalities compared to their wild-type littermates, demonstrating that PCDH7 is dispensable for survival. These data are consistent with DepMap data, which indicate that PCDH7 is not an essential gene (*43*). A more detailed evaluation of mAb7’s ability to direct immune surveillance will also be important for its use as an immunotherapeutic.

While our findings provide strong preclinical evidence for PCDH7-targeting antibody therapy, several limitations should be acknowledged. First, our studies primarily used xenograft and syngeneic tumor models, which may not fully recapitulate the complexity of human LUAD developing in its native TME. Although we used both subcutaneous and orthotopic models, autochthonous tumor models developing within the lung parenchyma would provide additional validation. Second, our characterization focused on LUAD, and the therapeutic potential of PCDH7 antibodies in other PCDH7-expressing malignancies, including pancreatic and prostate cancers, requires investigation. Third, while we demonstrated efficacy in adagrasib-resistant cells with elevated PCDH7 expression, the mechanisms by which tumors might develop resistance specifically to PCDH7 antibody therapy were not comprehensively explored. Long-term treatment studies examining potential resistance mechanisms and optimal dosing schedules will be critical for clinical development. Fourth, although we identified EC1 as the critical binding domain and demonstrated its functional importance for MAPK activation, the complete molecular mechanisms by which PCDH7 activates downstream signaling pathways and the specific immune cell subsets mediating ADCC warrant further investigation. Additional studies are warranted to assess the TME of Hu-mAb7–treated tumors. Given that different organs have distinct immune cell populations, it will be important to evaluate the TME in native lung tumors treated with anti-PCDH7 therapy. In addition, recent studies indicate that PCDH7 expression strongly correlates with immunologically cold tumors. In a mouse model of pancreatic ductal adenocarcinoma, PCDH7 expression, as evaluated by single-cell RNA-seq and IHC, significantly correlated with poor intratumoral CD8^+^ T cell infiltration (*16*). Consistent with this, CIBERSORT analysis revealed a negative correlation between PCDH7 and tumor infiltration by CD8^+^ T cells and activated NK cells in NSCLC (*15*). Thus, anti-PCDH7 therapy may help activate immunosurveillance in these settings. Last, we acknowledge that different genomic contexts may influence the therapeutic sensitivity. As such, it will be important to investigate antibody efficacy in the context of additional comutations in future studies.

In summary, these studies provide an important first step toward the clinical development of PCDH7-targeting antibodies. In the future, it may be possible to enhance the antitumor efficacy of mAb7 by generating a bispecific mAb7 (bsAb). Bispecific antibodies are a particularly exciting area in oncology because they simultaneously target tumor-associated antigens and engage cytotoxic immune cells (*44*). For example, BiTE (bispecific T cell engager) and BiKE (bispecific -cell engager) modifications typically target CD3 and CD16, respectively, along with an oncoprotein, thereby increasing the recruitment of TILs and enhancing a more efficient immune cell response to the targeted tumors (*45*, *46*). bsAb modifications can also overcome challenges such as crossing the blood-brain barrier. For example, bsAbs that target the transferrin receptor can significantly enhance antibody accumulation in the brain (*47*, *48*). The transferrin receptor–mediated blood-brain barrier shuttle for the delivery of antibody therapy in the brain is of particular interest given the important role that PCDH7 plays in promoting brain metastasis (*14*). Last, mAbs may also be engineered to deliver chemotherapeutic agents to tumors (*49*, *50*). Antibody-drug conjugates are a rapidly growing avenue for cancer therapeutics, with more than 150 antibody-drug conjugates currently in clinical trials (*50*). These therapeutics combine the high specificity of antibodies with the potent cytotoxic effects of chemotherapeutics to precisely eradicate tumors in synergy with the immune system. These efforts provide a promising path to the clinical development of PCDH7-targeting antibodies for the treatment of KRAS-mutant lung cancers and potentially other tumor types with high PCDH7 expression.

## MATERIALS AND METHODS

### Rabbit immunization and single memory B cell isolation

Human PCDH7 protein (ECDs) was used for antibody generation and was expressed in insect cells. The protein has a 6×HIS-tag (at the N terminus) and was purified to >95% purity using Ni-NTA resin (Sino Biologicals). To generate antibodies from immunized rabbits, two rabbits (NZW, Charles River) were immunized with the recombinantly produced PCDH7 using a series of immunization (three boost injections after primary priming) procedures. Titers of anti-PCDH7 sera were determined by a series of serum dilutions in ELISA for binding to PCDH7 protein coated on 96-well plates (high-binding plates, Thermo Fisher Scientific). When serum titer reached >10^6^, peripheral blood samples were collected from the immunized rabbits for B cell isolation from the freshly prepared PBMCs using a FACS instrument (BD FACSAria III, BD Biosciences). Sorted single B cells were collected in 96-well cell culture plates (Thermo Fisher Scientific) and cultured for 7 to 10 days in a cell culture incubator with 5% CO_2_ and 95% humidity in RPMI culture media with 10% fetal bovine serum (FBS), and cytokines and growth factors were added for enhanced antibody expression. The antibodies in the culture supernatants were screened for binding to PCDH7. Cells from the positive-binding wells were used to clone antibody sequences. Total RNA was isolated from individual positive B cell cultures, and cDNA was synthesized using a Superscript reverse transcriptase II (Invitrogen) according to the manufacturer’s suggestion. DNA sequences of antibody variable regions from both heavy chains and light chains were amplified by PCR using a set of primers and cloned into a vector for sequencing and antibody expression.

### Phage display antibody panning

PCDH7 ECD proteins were coated on high-binding tubes for capture (3-ml vial), and binding antibodies were displayed on phage. After three rounds of panning selection with increased washing stringency, phages with antibody binders were rescued by infecting TG1 *Escherichia coli* cells in culture. PCDH7 binding antibody hits in *E. coli* were screened by ELISA. Plasmid DNA from positive *E. coli* clones in ELISA was isolated and sequenced. Confirmed scFv antibody sequences were constructed for expression of full-length antibodies using a mammalian expression vector system in HEK293 cells (Invitrogen). Antibodies were purified using protein A affinity resin using fast protein liquid chromatography.

### Mice

NSG (NOD.Cg-*Prkdc^scid^ Il2rg^tm1Wjl^/*SzJ) mice, NSG-SGM3 [NOD.Cg*Prkdc^scid^Il2rg^tm1Wjl^* Tg (CMV-IL3,CSF2,KITLG)1Eav/MloySzJ], and athymic nude (NU/J) mice were ordered from The Jackson Laboratory (RRID: IMSR_JAX:005557, IMSR_JAX:013062, and IMSR_JAX:002019). All procedures involving mice were approved by the Institutional Animal Care and Use Committee of UTSW Medical Center.

### Cell lines

CDK4/TERT–immortalized HBEC3 (HBEC3-KT) cells with stable inhibition of TP53 and ectopic expression of KRAS^G12V^ (HBEC shp53;KRAS^G12V^) were provided by J. Minna (University of Texas Southwestern Medical Center, Dallas, TX) and maintained in complete KSFM growth medium (keratinocyte-free media; Gibco, 17005042) supplemented with 1% antibiotic-antimycotic (Thermo Fisher Scientific, 15240062), bovine pituitary extract (50 mg/ml), and epidermal growth factor (5 ng/ml; supplied with medium) (*51*). HBECs with enforced expression of PCDH7-V5 (HBEC shp53-*KRAS^G12V^*;*PCDH7*-V5) or PCDH7ΔC1-V5 (HBEC-shp53-*KRAS^G12V^*;*PCDH7*ΔC1-V5) were generated by infecting cells with PCDH7-V5;GFP or PCDH7ΔC1-V5:GFP lentivirus (where GFP refers to green fluorescent protein) and sorting for GFP^+^ cells. H1944, PC9, DFCI024, HOP-62, H2030, HCC515, and H2887 cells were obtained from J. Minna and maintained in complete RPMI 1640 growth medium (RPMI 1640; Invitrogen, A1049101) and supplemented with 5% FBS (Sigma-Aldrich, F2442) and 1% antibiotic-antimycotic (*52*). DFCI024 AR cells were isolated from xenograft tumors by first mechanically dissociating the tumors and then incubating cells with trypsin-EDTA (0.05%) (Gibco, 25300054) before plating into complete RPMI 1640 supplemented with 5% FBS and 0.05 μM adagrasib. KP67-1 cells and KP cells were obtained from E. Akbay and D. McFadden, respectively, and maintained in complete RPMI 1640 growth media supplemented with 10% FBS. CMT167 cells (derived from a spontaneous lung tumor in C57BL/6 mice and containing an oncogenic *KRAS^G12V^* mutation) were cultured in RPMI 1640 media supplemented with 10% FBS and 1% antibiotic-antimycotic. PCDH7 depletion in H1944 cells was performed using Lenti-CRISPRv2 followed by selection with puromycin (2 μg/ml; Thermo Fisher Scientific, A1113803). All cell lines were cultured in a standard tissue culture incubator at 37°C with 5% CO_2_ and tested for mycoplasma by PCR before xenograft transplantation.

### Plasmids

LentiCRISPRv2 sg*PCDH7* and sgControl constructs used for PCDH7 depletion in the H1944 cell line have been previously described (*7*). PCDH7-V5: GFP and PCDH7ΔC1-V5: GFP lentiviral constructs were generated using VectorBuilder.

### In vitro screening of antibody candidates

For ICW and MTT antibody screening, 1 × 10^3^ LUAD cells were incubated with PCDH7-neutralizing antibody candidates (10 μg/ml) in complete RPMI 1640 growth medium. Each plate also included corresponding isotype control antibodies in triplicate at 10 μg/ml. The in-house–made rabbit IgG isotype control was used for rabbit B cell–derived antibodies, and the Ultra-LEAF Purified Human IgG1 Isotype Control Recombinant Antibody (BioLegend, 403501) was used for phage display antibodies. Cells were cultured at 37°C with 5% CO_2_ for 96 hours before analysis. For MEK inhibitor combination studies, cells were treated with 0.02 μM trametinib (Thermo Fisher Scientific, NC0596487). For KRAS inhibitor combination studies, cells were treated with 0.05 μM adagrasib (Selleck Chemicals, S8884). ERK phosphoactivation was assessed by ICW assay. Growth media were aspirated, and cells were immediately fixed for 15 min with 3.7% formaldehyde (Electron Microscopy Sciences, 15710) in tris-buffered saline (TBS). Samples were washed and permeabilized three times with Triton wash buffer (TBS + 0.2% Triton X-100) before blocking with LI-COR Intercept (TBS) Blocking Buffer (LI-COR, 927-60001) for 1.5 hours. Cells were incubated overnight with mouse anti–phospho-p44/42 MAPK (Cell Signaling Technologies, 9106L) diluted 1:50 in LI-COR Intercept (TBS) Blocking buffer and 0.2% Triton X-100 at 4°C with gentle rocking. Following primary antibody incubation, samples were washed five times with Tween wash buffer (TBS + 0.1% Tween 20) and then incubated for 2 hours with IRDye-800CW–conjugated goat anti-mouse secondary antibody (LI-COR, 926-32210) diluted 1:800 and CellTag 700 Stain diluted 1:500 (LI-COR, 926-41090) in LI-COR Intercept (TBS) Blocking buffer. Samples were washed five times with Tween wash buffer and then once with TBS before imaging with the LI-COR Odyssey DLx system. All washes were performed for 5 min each with gentle rocking at room temperature. Cell viability was measured using the CellTiter 96 nonradioactive cell proliferation assay (Promega, G4000) according to the manufacturer’s instructions.

### Xenograft assays

Lung cancer cells (1 × 10^6^) were injected subcutaneously into the right flanks of 4-week-old NSG mice, 4-week-old NU mice, or 16-week-old humanized mice in RPMI 1640 without supplement. Following the formation of palpable tumors (tumor volume, ~20 mm^3^), mice were randomized into equal groups and treated once per week with the designated antibodies (5 to 10 mg/kg) in phosphate-buffered saline via intraperitoneal injection. For trametinib combination experiments, mice received daily trametinib vehicle [0.5% hydroxypropylmethyl cellulose and 0.2% Tween 80 in ddH_2_O (double-distilled water)] or trametinib at 1 mg/kg via oral gavage. For adagrasib combination experiments, mice received daily adagrasib vehicle [5% DMSO (dimethyl sulfoxide), 30% PEG-300 (polyethylene glycol, molecular weight 300), 5% Tween 80, and 50% ddH_2_O] or adagrasib at indicated concentrations via oral gavage. Tumors were measured using a caliper every 3 days until the end point. Tumor volume was calculated using the formula (length × width^2^)/2. Where indicated, blood serum was collected 7 days after the first antibody injection and at the experimental end point.

### RNA extraction and RNA-seq

Tumor tissue chunks (30 to 50 mg) were homogenized using a polytron, and RNA was extracted using TRIzol (Ambion by Life Technologies, 15596018). Extracted RNA was cleaned up by the RNeasy Mini Kit (Qiagen, 74104) and then sequenced at the McDermott Center Next Generation Sequencing Core at UT Southwestern (UTSW) Medical Center. GSEAs, functional overrepresentation analyses, and Ingenuity Pathway Analyses were performed at the McDermott Center Bioinformatics lab. Pathway and network analyses were conducted using GSEA software and Qiagen’s IPA Analysis Match tool (Qiagen, 836508). For GSEA, all genes were ranked with logCPM > 0 and threshold FDR (false discovery rate) < 0.25. Functional overrepresentation analysis was performed using WebGestalt 2019 (*53*) with the Gene Ontology (Biological Process), Kyoto Encyclopedia of Genes and Genomes, and REACTOME databases using thresholds FDR < 0.05 and logCPM >0.

### Immunohistochemistry

IHC staining for Ki67 and p-ERK1/2 was performed as previously described using anti-Ki67 [Cell Signaling Technology (CST), no. 9027] or anti–phospho-p44/42 MAPK (CST, no. 9101) antibodies (*10*). TUNEL assays on formalin-fixed paraffin-embedded tumor tissue sections were performed using the DeadEnd Fluorometric TUNEL System (Promega, G3250) according to the manufacturer’s instructions. Slides were mounted using Vectashield Hardset antifade mounting medium (Vector Laboratories, H-1400). IHC-F staining for CD3ε (CST, no. 39714), CD8α (CST, no. 47865), and GrzB (CST, no. 34103) was performed according to the manufacturer’s instructions for the SignalStar Miniplex IHC kit (CST, no. 63043S). Stained slides were scanned using the Zeiss Axioscan 7 scanner and visualized using Zen Microscopy software (version 3.10).

### Ki67, p-ERK1/2, TUNEL, and GrzB index

Ki67-positive and phospho–p44/42 MAPK (p-ERK1/2)–positive cells were quantified by counting brown nuclei and hematoxylin (blue) counterstain. TUNEL-positive cells were quantified by counting green nuclei and 4′,6-diamidino-2-phenylindole (DAPI) counterstain. CD8^+^ T cells were quantified by counting DAPI^+^CD3^+^CD8^+^ cells. GrzB^+^CD8^+^ T cells were quantified by counting DAPI^+^CD3^+^CD8^+^GrzB^+^ cells. The Ki67, p-ERK1/2, and TUNEL index for each mouse was calculated as percent positive cells = number of positive nuclei/total cell nuclei × 100, as described previously (*54*). The GrzB index (represented as %GrzB^+^ CD8 T cells) was calculated as follows: percent GrzB-positive cells = number of GrzB-positive CD8 T cells/total CD8 T cells × 100. For Ki67 quantification, *n* = 2 animals per group were analyzed, and five independent fields per animal were quantified. For p-ERK1/2 quantification, *n* = 3 animals per group were analyzed, and 10 independent fields were quantified per animal. For TUNEL and GrzB quantification, *n* = 3 animals per group were analyzed, and 10 independent fields were quantified per animal.

### Humanized mice and human cord blood sample processing

Four-week-old female NSG or NSG-SGM3 recipient mice were given enrofloxacin in water ad libitum beginning 3 days before humanization and continuing for 2 weeks after humanization. Mice were irradiated with 100 centigrays (x-ray irradiation with an X-RAD320 irradiator) to ablate bone marrow 6 hours before engraftment. Heparinized human cord blood samples were obtained from UTSW Parkland Hospital in accordance with the regulations and guidelines for using human cord blood at UTSW (Institutional Review Board: STU 112010-047). Human granulocytes were isolated by Ficoll-Paque PLUS (Cytiva, 17144003) density gradient centrifugation according to the manufacturer’s instructions. Human CD34^+^ cells were further purified using the EasySep Human CD34 Positive Selection kit (STEMCELL Technologies, catalog no. 17856) according to the manufacturer’s instructions and resuspended in phosphate-buffered saline. CD34^+^ cells (5 × 10^4^) were injected intravenously into recipient mice. Twelve weeks after engraftment, humanization was validated by flow cytometry analysis of peripheral blood. Humanized mice with >30% human CD45^+^ cell reconstitution were included in xenograft tumor studies. Human cord blood samples were obtained from UTSW Parkland Hospital in compliance with the regulations and the use approval of human cord blood (STU 112010-047) at UTSW Medical Center. Sterile blood was obtained at the time of cesarean section from deidentified human umbilical cords that are normally discarded. The procedure was approved under a protocol exempt from informed consent as approved by the Institutional Review Board of UTSW and the Office for Human Research Protections, supported by the US Department of Health and Human Services. To maintain anonymity, links between the donor’s medical and social histories, including fetal sex, are not maintained.

### Orthotopic transplantation

Six- to eight-week-old female humanized NSG mice were transplanted with 0.3 × 10^6^ luciferase-expressing H1944 lung cancer cells in serum-free medium containing 50% Matrigel. Mice were anesthetized with a 2.5% isoflurane and oxygen mixture. Cells were injected ~1.5 cm above the lower left rib line through the intercostal region. Mice were then placed on a heating pad and observed until they revived from anesthesia. Mice were randomized and treated with either control or Hu-mAb7 (10 mg/kg) by weekly intraperitoneal injection. Tumor growth was monitored once per week using bioluminescence imaging (AMI-HTX system, Spectral Instruments) after administration of luciferin (Gold Biotechnology, catalog no. LUCK-2G). Photons per second values were generated using Spectral Instruments Aura 4.0 software. Body weights were recorded weekly.

### ADCC assay

ADCC activity mediated by Hu-mAb7 was detected in a real-time cytotoxicity assay as described previously (*55*) using xCELLigence instrument (ACEA Biosciences, San Diego, CA). Briefly, PC9 LUAD cells with overexpression of PCDH7 were used as target cells and seeded in E-plate 96 (ACEA Biosciences, Inc., San Diego, CA). Human PBMCs, which were used as immune effector cells, were added for a final effect:target ratio of 50:1. Antibodies were added at 2 μg/ml, and the cell index (cell growth) was monitored continuously for 3 days. The cell index recorded after treatment with Hu-mAb7, LALAPG, and humanized control antibodies was used to calculate the percentage of cell lysis using the formula (Cell index of control group − cell index of treatment group)/(cell index of control group) × 100.

### GrzB ELISA

The experimental protocol for coculture was adapted from Kim *et al.* (*56*). The protocol for stimulation of GrzB was adapted from Efimova and Kelly (*38*). Briefly, 5 × 10^4^ H1944 cells or PC9 cells with overexpression of PCDH7 were cultured with the indicated antibody (10 μg/ml) in complete RPMI at 37°C with 5% CO_2_ overnight. The next day, the media were removed and replaced with 4 × 10^5^ NK-92 MI cells or Jurkat T cells in 250 μl of Jurkat T cell media (complete RPMI 1630 growth medium with 10% FBS). For Jurkat T cells, activating antibodies [final concentrations: CD3 (2 μg/ml; Thermo Fisher Scientific, 16-0037-81), CD28 (1 μg/ml; Thermo Fisher Scientific, 16-0289-81), and interleukin-2 (0.0178 μg/ml; Abcam, ab9619)] were added to each well in an additional 50 μl of Jurkat T cell media after 1 hour. After 24 hours of culture, supernatants were collected and centrifuged to clarify. The GrzB concentration in the supernatant was assayed using the Abcam Human Granzyme B ELISA Kit according to the manufacturer’s instructions (Abcam, ab235635).

### Mass CyTOF flow cytometry analysis of TILs

Tumors were excised from euthanized mice and homogenized using a gentleMACS Octo Dissociator (Miltenyi Biotec, 130-096-427). Tumor cells were digested at 37°C at 150 rpm for 30 min in digestion buffer [RPMI 1640 containing 10% FBS, collagenase A (1 mg/ml; Sigma-Aldrich, 10103578001), and deoxyribonuclease I (5 μg/ml; Sigma-Aldrich, 10104159001)]. All subsequent steps were performed on ice. Digested tumors were filtered through a 70-μm cell strainer, pelleted at 500*g*, and incubated with Red Blood Cell lysis buffer (Sigma-Aldrich, 11814389001). Cells were washed twice and then processed for Helios Mass CyTOF (Fluidigm) according to the manufacturer’s instructions. Antibodies are listed in table S3. Briefly, cells were stained with cisplatin (5 μM) for 5 min at room temperature, quenched with Maxpar Cell staining buffer (Fluidigm), washed, and counted. One to three million cells were analyzed per sample. The cells were centrifuged and resuspended in 50 μl of Fc Receptor Blocking Solution, incubated for 10 min at room temperature, and then incubated with the cell surface antibody cocktail for 30 min at room temperature. Cells were washed and centrifuged twice, fixed with 1× Maxpar Fix I Buffer for 30 min at room temperature, washed twice, and resuspended and incubated in the cytoplasmic/secreted antibody cocktail for 30 min. Cells were washed and centrifuged twice and then incubated with Invitrogen FOXP3 Fixation/Permeabilization solution for 30 min, followed by two more washes and incubation with the nuclear antigen antibody cocktail for 30 to 45 min. Cells were then washed and fixed with a fresh 1.6% formaldehyde solution for 10 min, then centrifuged, and stained overnight with 125 nM Cell-ID Intercalator-Ir at 4°C. The next day, cells were washed twice with Maxpar Cell Acquisition Solution, filtered through a nylon membrane into flow cytometry tubes for analysis, and pelleted. Samples were analyzed at the UTSW Flow Cytometry Core Facility (Helios, Standard BioTools). FlowJo software was used to gate live, single cells. OMIQ was then used to calculate immune cell populations (%) of CD45^+^ live cells.
