# Supplementary material for: Monoclonal antibodies targeting PCDH7 inhibit tumor growth and enhance immune responses in KRAS-mutant non–small cell lung cancer
Source: Sci Adv. 2026 Jun 3;12(23):eaeb0794. doi: 10.1126/sciadv.aeb0794 (PMC13232558; doi:10.1126/sciadv.aeb0794)
Supplement: Supplementary file 1 — Figs. S1 to S8 Table S1 Legends for tables S2 and S3 [file sciadv.aeb0794_sm.pdf]

Science Advances
AAAS

Supplementary Materials for

Monoclonal antibodies targeting PCDH7 inhibit tumor growth and enhance immune responses in *KRAS*-mutant non–small cell lung cancer

Nicole Novaresi *et al.*

Corresponding author: Zhiqiang An, zhiqiang.an@uth.tmc.edu; Ningyan Zhang, ningyan.zhang@uth.tmc.edu; Kathryn A. O'Donnell, kathryn.odonnell@utsouthwestern.edu

Sci. Adv. **12**, eaeb0794 (2026)
DOI: 10.1126/sciadv.aeb0794

The PDF file includes:

Figs. S1 to S8
Table S1
Legends for tables S2 and S3

Other Supplementary Material for this manuscript includes the following:

Tables S2 and S3

Figure S1

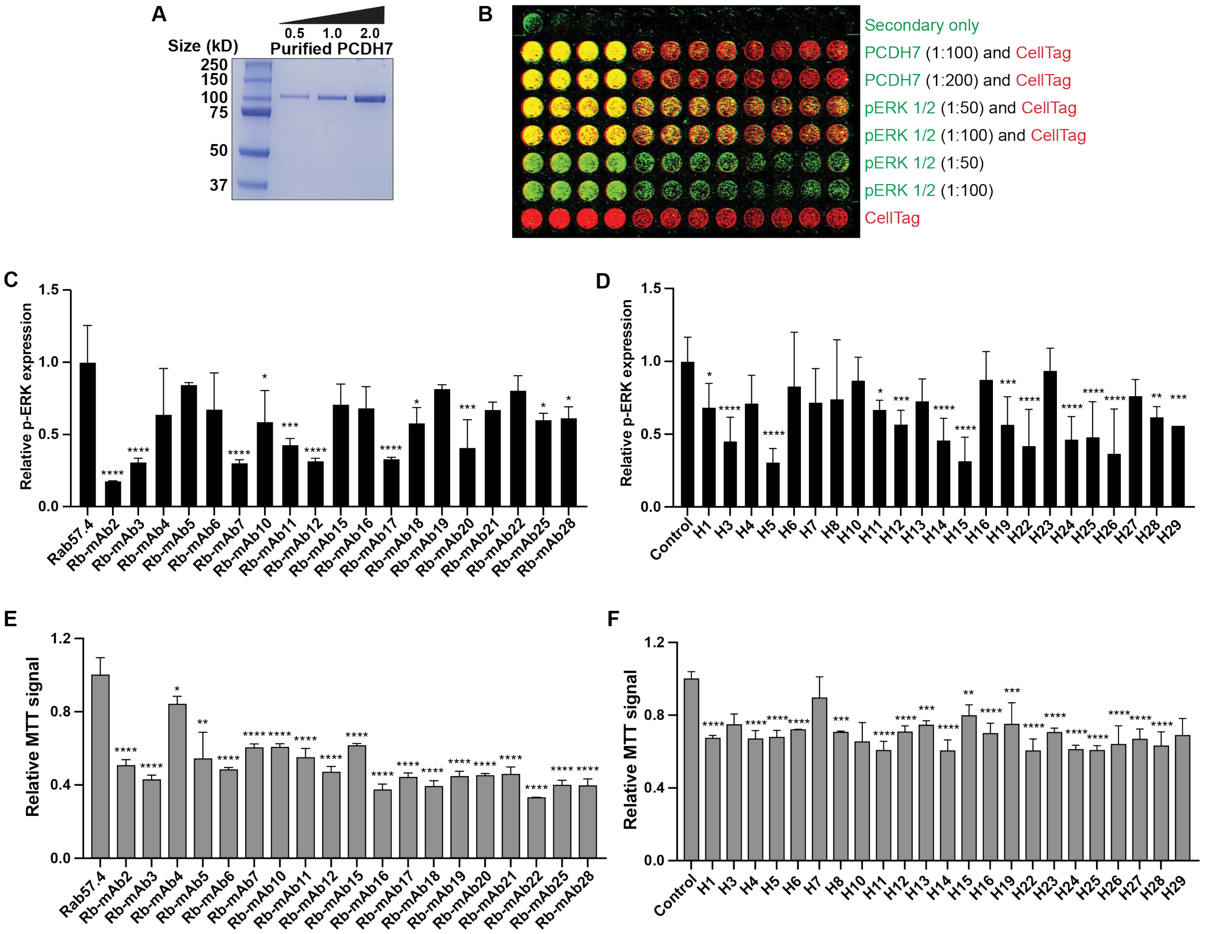

Supplementary Figure S1. Generation of anti-PCDH7 mAbs. A, Coomassie gel of purified PCDH7 protein (the extracellular domain was expressed in insect cells with a polyhistidine tag at the N-terminus). **B,** Representative image of LiCOR in-cell western assay for phospho-ERK. KRAS mutant H1944 cells were probed overnight with anti-pERK primary antibody before labeling with IRDye-800CW-conjugated goat anti-rabbit secondary antibody and CellTag 700 Stain. **C,D,** Quantification of pERK values (IRDye-800) following 96h treatment with 10ug/ml purified monoclonal B cell antibodies (**c**) or phage display antibodies (**D**) normalized to CellTag 700 and reported relative to control antibody values. Experiments were repeated twice for confirmation. **E,F,** Quantification of MTT values following 96hr treatment with 10ug/ml purified monoclonal B cell antibodies (**E**) or phage display antibodies (**F**) reported relative to control antibody values. Error bars indicate standard deviation and n=3. Experiments were repeated twice for confirmation.

Figure S2

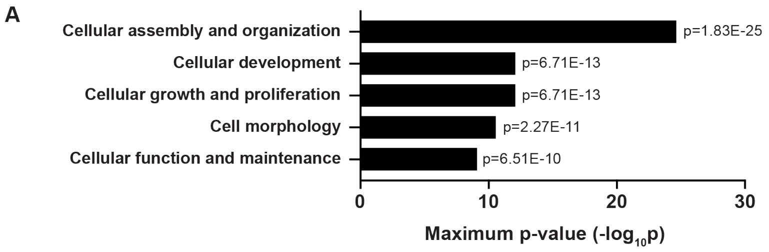

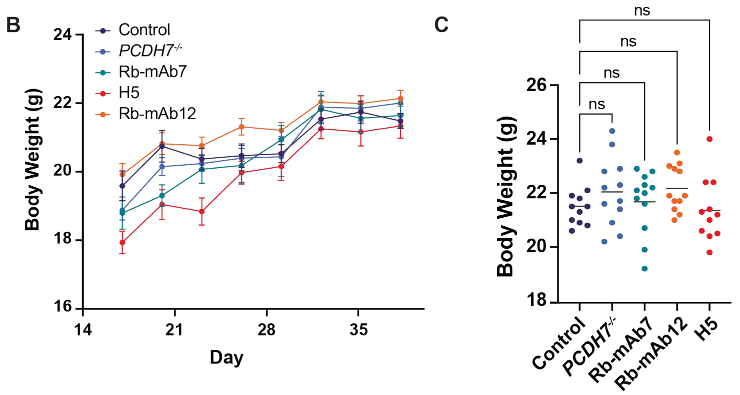

D

	Serum Antibody (μg/mL)	
**Treatment**	**Day 20**	**Day 38**
Rb-mAb7	14.3	27.09
Rb-mAb12	14.11	61.06
H5	8.62	21.66

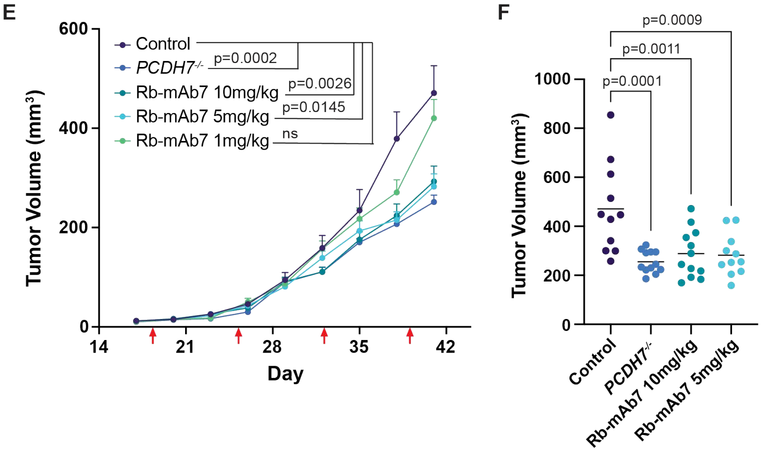

Supplementary Figure S2. RNA-seq, body weight, serum analyses, and dosage studies. A, Ingenuity pathway analysis (IPA) of Control vs Rb-mAb7 RNAseq data for molecular and cellular functions. Bars represent maximum -log10p values from analysis, and p-values are depicted next to each biological function. **B,** ELISA data showing concentration of indicated antibody in blood sera collected at day 20 and endpoint (day 38) from tumor-bearing mice (tumor volumes in **Fig. 2A**). Data are reported after subtracting background values determined from sera of untreated mice bearing H1944 PCDH7$^{-/-}$ tumors. **C,** Body weights of tumor-bearing mice (tumor volumes in Fig 2A) treated with 10 mg/kg of the indicated antibodies measured every 3

days until endpoint at day 38. **D,** Final body weights of mice from (**C**). Statistics were generated by one-way ANOVA with Dunnett's multiple comparisons. Error bars indicate standard deviation. **E,** Tumor volumes of H1944 or H1944 PCDH7$^{-/-}$ cells treated weekly with 10 mg/kg control antibody or Rb-mAb7 at the indicated concentrations in immunocompromised NSG mice (n=11 for all groups) measured every 3 days until endpoint at day 41. **F,** final tumor volumes from **E**.

Figure S3

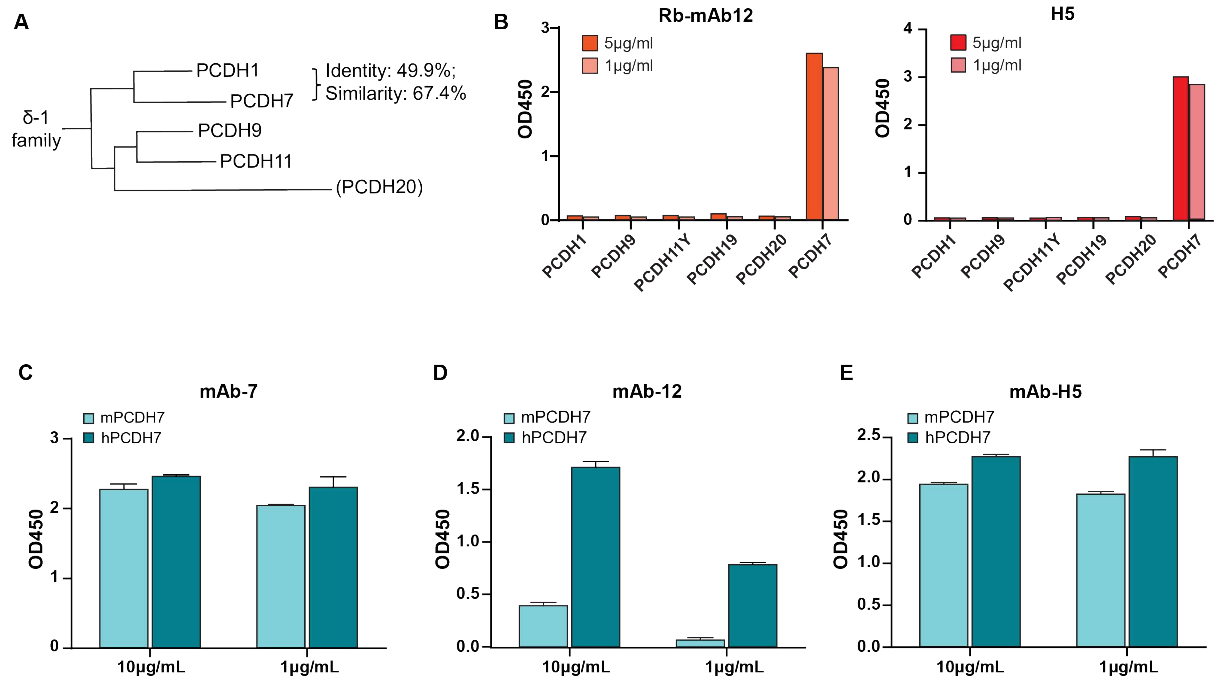

Supplementary Figure S3. Binding specificity of Rb-mAb7. A, Schematic depicting homology between PCDH7 and other δ-1 protocadherin family members. **B,** Evaluation of Rb-mAb12, Rb-mAb17, and H5 PCDH7 antibody cross-reactivity to δ-1 protocadherin family members by ELISA. **C,D,E.** Evaluation of PCDH7 antibody cross-reactivity to murine PCDH7.

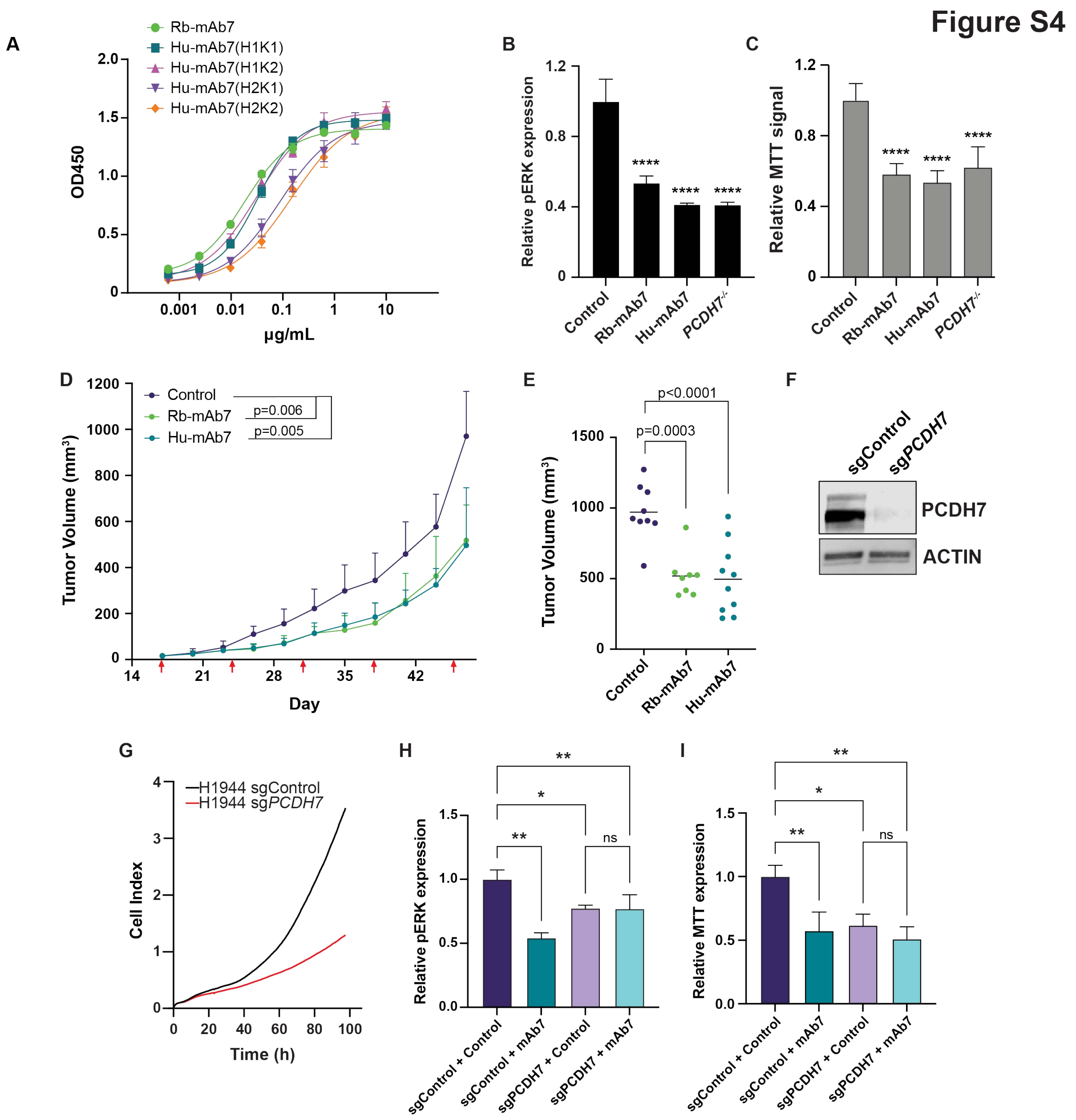

Supplementary Figure S4. Testing the efficacy of humanized mAb7. A, Binding affinity of humanized mAb7 clones with different heavy and light chains using antibody titration ELISA. Hu-mAb7 (H1K1) is shown in **Figure 4B**. **B-C,** Quantification of phospho-ERK (**B**) and MTT values (**C**) following 96-hour treatment of H1944s with 10ug/ml of the indicated antibody, reported relative to control antibody values. Untreated *PCDH7*-/- cells were included as positive controls for each experiment. Experiments were repeated twice for confirmation. **D,** Tumor volumes of H1944 cells treated weekly with 10 mg/kg of the indicated antibodies in

immunocompromised NSG mice (n=9 control, n=10 Rb-mAb7, n=11 Hu-mAb7) measured every 3 days until endpoint at day 47. Statistics were generated by generalized linear mixed models (GLMM). **E**, final tumor volumes from **D**. **F,** Validation of PCDH7 protein expression in Cas9-expressing sgControl and sg*PCDH7* H1944 cell lines. **G**, Comparative cell growth between in H1944 sgControl and H1944 sg*PCDH7* cell lines. **H, I,** Quantification of phospho-ERK (**H**) and MTT values (**I**) following 96hr treatment of H1944 sgControl and H1944 sg*PCDH7* cells with 10ug/ml of the indicated antibody, reported relative to control-treated H1944 sgControl values. Statistics were generated by one-way ANOVA with Dunnett's test for multiple comparisons, ** = $p<0.01$, * = $p<0.05$. Experiments were repeated twice for confirmation.

Figure S5

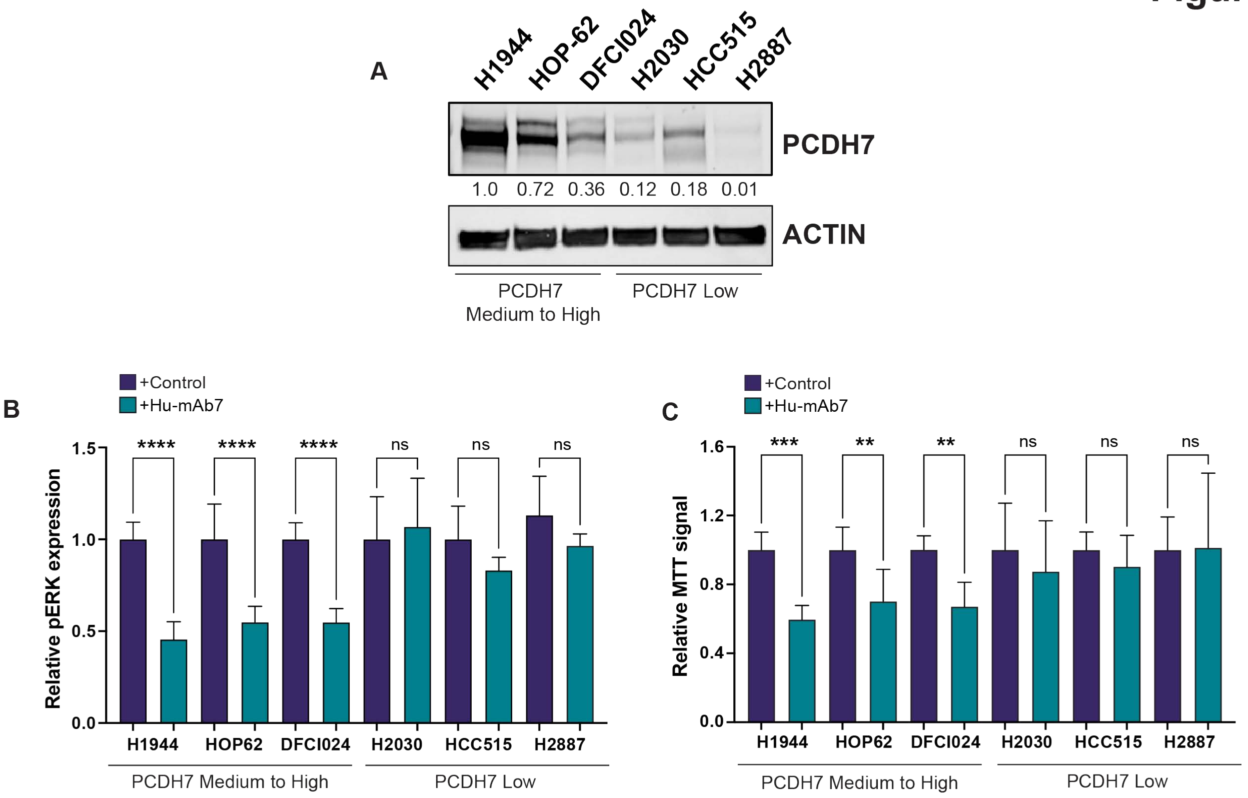

Supplementary Figure S5. Hu-mAb7 efficacy depends on PCDH7 expression. A, PCDH7 protein expression for "PCDH7 Medium to High" and "PCDH7 Low" tumors by western blot. Quantification indicates each cell line's PCDH7 expression normalized to ACTIN, then presented relative to that value for the H1944 cell line. **B, C,** In-cell western quantification of phospho-ERK expression (**B**) and MTT values (**C**) for cell lines treated with Control and Hu-mAb7 antibodies. Values were reported relative to each cell line's control-treatment values. Statistics were generated by one-way ANOVA with Dunnett's test for multiple comparisons, **** = $p<0.0001$, *** = $p<0.001$, ** = $p<0.01$. Experiments were repeated twice for confirmation.

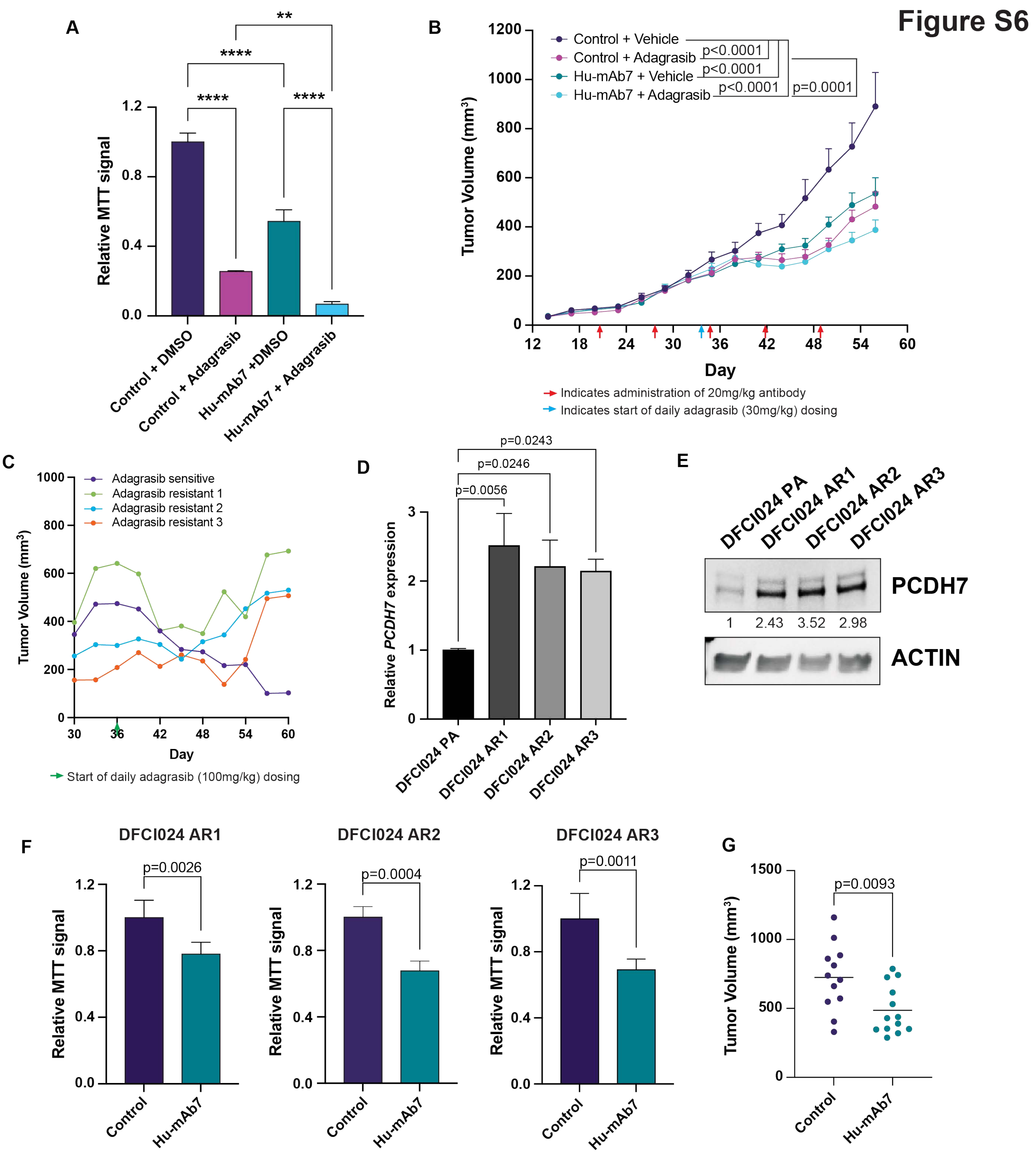

Supplementary Figure S6. Assessing efficacy of Hu-mAb7 combination therapy with the *KRAS*^{G12V} small molecule inhibitor adagrasib. A, MTT assay data showing cell viability in DFCI024 cells treated with the

indicated treatments. MTT signal was reported relative to control+DMSO values. Statistics were generated by one-way ANOVA with Dunnett's test for multiple comparisons, **** = $p<0.0001$, ** = $p<0.01$. Experiments were repeated twice for confirmation **B,** Tumor volumes of mice harboring DFCI024 xenografts (*n*=13-14) treated with 20 mg/kg of the designated antibody with or without daily adagrasib (30mg/kg) measured every 3 days over 57 days. Red arrows denote antibody treatments (once per week), and the blue arrow indicates the start of daily adagrasib or vehicle administration. Statistics were generated by generalized linear mixed models (GLMM). **C,** Growth curve of the tumor used to generate DFCI024 AR cell line, with growth curve of an adagrasib-sensitive tumor for comparison. **D,E,** PCDH7 mRNA (**D**) and protein (**E**) expression in DFCI024 parental cell line (DFCI024 PA) compared to DFCI024 adagrasib-resistant (DFCI024 AR) cell line. **F,** MTT assay data showing cell viability in DFCI024 AR cells treated with control or Hu-mAb7 antibodies. MTT signal was reported relative to control values. **G,** Final volumes (day 30) of DFCI024 AR xenograft tumors treated with control or Hu-mAb7 antibodies (*n*=12-13). Statistics for **D, F,** and **G** were calculated by an unpaired t-test.

Figure S7

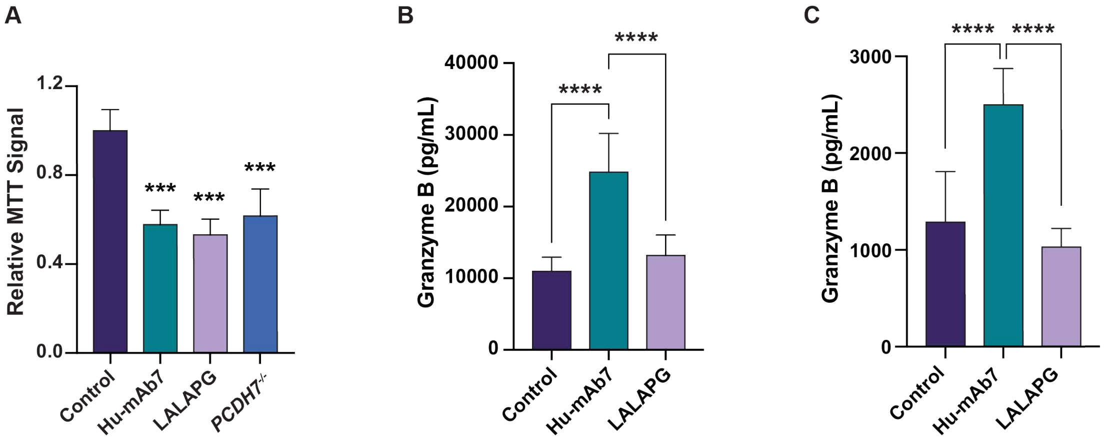

Supplementary Figure S7. Validating the Fab efficacy of Hu-mAb7 LALAPG antibody and cytotoxic efficacy of Hu-mAb7. A, MTT assay data showing cell viability in control-, Hu-mAb7-, or Hu-mAb7 LALAPG-treated cells, or PCDH7-/- cells. Error bars indicate SD and n=3. Experiments were repeated twice for confirmation. **B, C,** Granzyme B ELISA assay for NK-92 MI cells (**B**) or activated Jurkat T cells (stimulated with CD4, CD28, and IL-2 functional antibodies) (**C**) co-cultured for 24 hours with H1944 cells and treated with the indicated antibodies. Statistics were generated by one-way ANOVA.

Figure S8

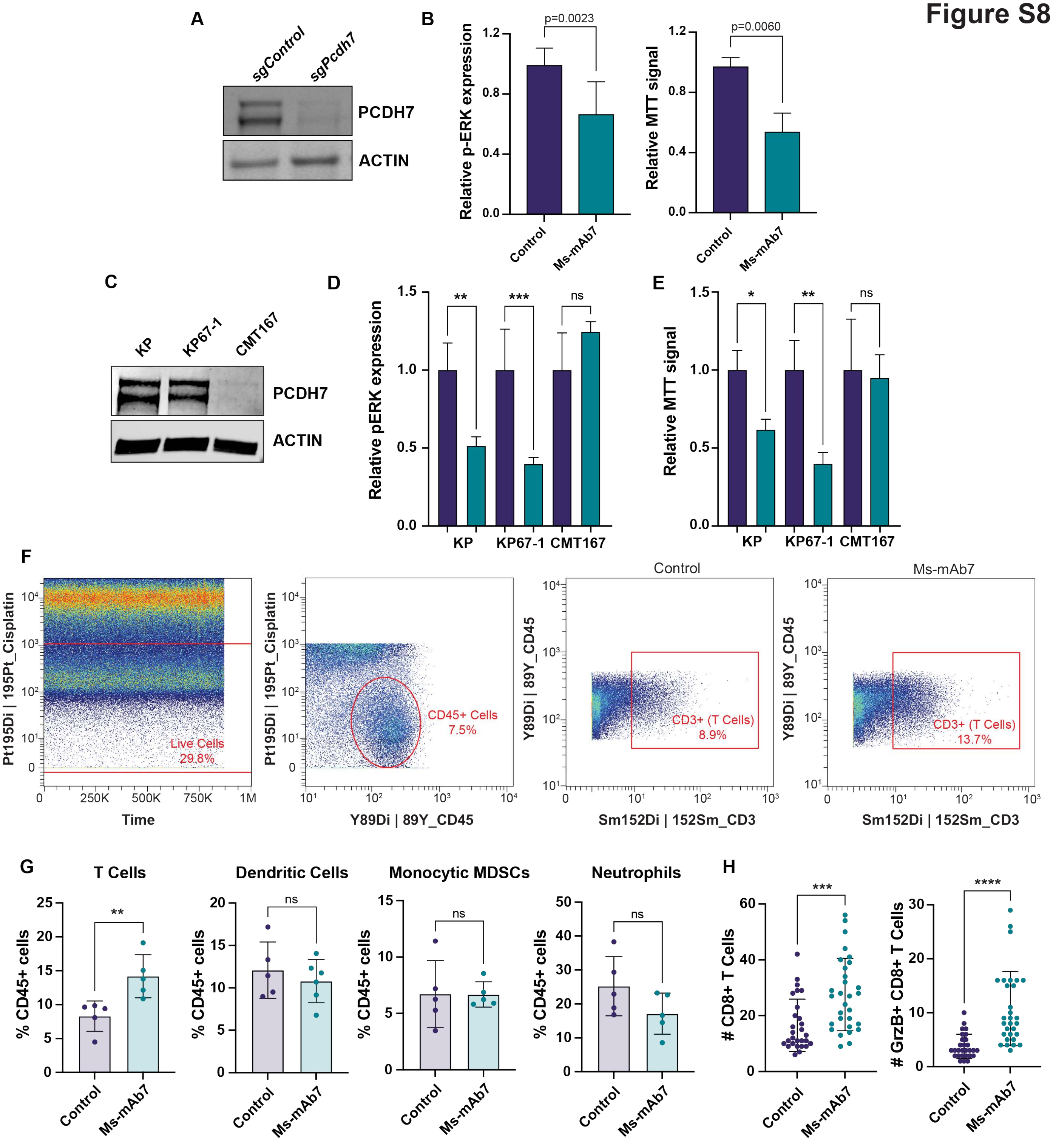

Supplementary Figure S8. Chimeric mAb7 reduces cell viability and enhances immune response in a syngeneic *KRAS*^{G12D} tumor model. A, Validation of PCDH7 expression in KdP67-1 cells by western blot. **B,** Quantification of phospho-ERK and MTT values for cells treated with isotype control or mouse chimeric

mAb7 (Ms-mAb7) antibodies. Values reported relative to isotype controls, and statistics were generated for both assays by unpaired t-test. **C,** PCDH7 protein expression in murine LUAD cell lines. **D, E,** In-cell western quantification of phospho-ERK expression (**D**) and MTT values (**E**) for cell lines treated with Control and Ms-mAb7 antibodies. Values were reported relative to each cell line's control-treatment values. Statistics were generated by one-way ANOVA with Dunnett's test for multiple comparisons, *** = $p<0.001$, ** = $p<0.01$, * = $p<0.05$. Experiments were repeated twice for confirmation. **F,** T cell gating strategy as an example for mass CyTOF analysis. Live cells were identified by excluding cisplatin+ events. Live immune cells were distinguished using $CD45^{+}$, then further gated for $CD3^{+}$ to identify T cells. **G,** Quantification of cell types identified (in addition to those shown in the main text figure **5E**) by mass CyTOF analysis. The following markers were used for each cell type: Dendritic cells ($CD11b^{+}CD11c^{+}IA/IE^{+}$), Monocytic MDSCs ($CD11b^{+}Ly\text{-}6C^{+}Ly\text{-}6G^{-}$), Neutrophils ($CD11b^{+}Ly\text{-}6G^{+}IA/IE^{-}$). **H,** Quantification of cytotoxic T cells ($CD3^{+}CD8^{+}$) and granzyme B^{+} cytotoxic T cells ($CD3^{+}CD8^{+}GrzB^{+}$). Values used to calculate the percentage of granzyme B^{+} cytotoxic T cells (main text figure **5G**).

Supplementary Table S1. Estimated kinetic binding constant (KD) of PCDH7 mAbs determined using BLI based Octet (96-Red) instrument.

**mAb Name**	**KD (M)**	**K_{on} (1/Ms)**	**K_{dis} (1/s)**	**Full X^2**	**Full R^2**
PCDH7-H5 (mAb-H5)	1.09E-09	1.23E+05	1.34E-04	2.330	0.995
PCDH7-mAb7	5.36E-10	9.86E+04	5.29E-05	0.458	0.999
PCDH7-mAb7Hu	6.08E-10	6.66E+04	4.05E-05	0.129	0.999
PCDH7-mAb12	1.74E-11	8.56E+04	1.49E-06	0.084	0.996
PCDH7-mAb17	2.79E-09	4.16E+05	1.16E-03	0.209	0.809

Supplementary Table S2. RNA-seq analysis. Downregulated gene sets in Rb-mAb7-treated xenograft tumors. *See the Excel spreadsheet.*

Supplementary Table S3. Antibodies used in this study. List of antibodies used for all experiments. *See the Excel spreadsheet*.
